# Folate receptor β performs an immune checkpoint function in activated macrophages

**DOI:** 10.3389/fimmu.2025.1638907

**Published:** 2025-09-29

**Authors:** Fenghua Zhang, Md Yusuf Al-Amin, Sagar Utturkar, Rina Jiang, Gregory Cresswell, Rami Alfar, Ian Ophaug-Johansen, Gabriel Bachman, Madduri Srinivasarao, Richard Finnell, Amaya Puig-Kröger, Timothy Ratliff, Philip S. Low

**Affiliations:** ^1^ Department of Chemistry and Institute for Drug Discovery, Purdue University, West Lafayette, IN, United States; ^2^ Purdue University Interdisciplinary Life Sciences Graduate Program, Purdue University, West Lafayette, IN, United States; ^3^ Purdue University Institute for Cancer Research, Purdue University, West Lafayette, IN, United States; ^4^ Department of Comparative Pathobiology, Purdue University, West Lafayette, IN, United States; ^5^ Department of Medicinal Chemistry and Pharmacognosy, Jordan University of Science and Technology, Irbid, Jordan; ^6^ Department of Agricultural and Biological Engineering, Purdue University, West Lafayette, IN, United States; ^7^ Center for Precision Environmental Health, Departments of Molecular and Human Genetics, Molecular and Cellular Biology and Medicine, Baylor College of Medicine, Houston, TX, United States; ^8^ Unidad de Immunometabolismo e Inflamación, Instituto de Investigación Sanitaria Gregorio Marañón, Hospital General Universitario Gregorio Marañón, Madrid, Spain

**Keywords:** immune checkpoint, macrophages, MDSCs (myeloid-derived suppressor cells), folate receptor beta (FRβ, Folr2), tumor-associated macrophages (TAMs), tumor microenvironment, cancer immunotherapy, macrophage polarization

## Abstract

Monocytes and macrophages are sentinels of the immune system that distinguish themselves from other cells by expressing the beta isoform of the folate receptor (FRβ). Because FRβ does not bind folate until the monocyte/macrophage is exposed to immunosuppressive cytokines, the question naturally arose whether FRβ might also perform an immune-related function. To examine this matter, we compared the properties of wild type (WT) and FRβ knockout mice. We observe that FRβ knockout (KO) mice display autoimmune symptoms that can include alopecia, enlarged spleens, and dermatitis, despite having normal cellular folate levels. We further demonstrate that syngeneic tumors (TRAMP C2, MC38) grow much slower in FRβ KO mice than wildtype mice. Comparison of cells extracted from syngeneic tumors of KO mice further reveal that CD69+ T cells are increased while PD1+ T cells and PD-L1+ myeloid cells are decreased in KO tumors. More detailed comparison of the bone marrow-derived macrophages from KO and WT mice demonstrates that KO mice have upregulated pro-inflammatory genes and downregulated anti-inflammatory genes. Because blockade of FRβ with a monoclonal antibody or deletion of FRβ impairs direct macrophage suppression of T cell activation *in vitro*, we conclude that FRβ performs a checkpoint function that regulates the immunologic properties of tumor myeloid cells. Since FRβ expression in human cancers is shown to correlate inversely with overall survival, we further posit that FRβ similarly performs an immunosuppressive function in human tumors.

## Introduction

Folates constitute a group of related B vitamins that are essential for the synthesis of nucleotide bases, several amino acids, and many other biochemical building blocks in which a methylation reaction is required (1). Cells that undergo rapid proliferation, enlargement, and/or repair therefore require substantial quantities of folates to support their anabolic functions (2). Because folates are not synthesized by mammals, they must be obtained in the diet and internalized into cells with the aid of folate transporters, primarily the reduced folate carrier (RFC) and the proton coupled folate transporter (PCFT) (3–5). Folate receptors, which are only expressed on a very few cell types (6), are not required for folate transport into most cells.

While the functions of RFC and PCFT are well established (5), the roles of folate receptors remain poorly understood. In the case of proximal tubule cells of the kidneys, folate receptor α (FRα) is required to capture folates in the urine and transport them against a concentration gradient back into the blood; i.e. in order to prevent folate deficiency (7). Similarly, FRα on the choroid plexus may assure that the high folate concentrations required in the brain can be generated from the low folate concentrations in the blood (8). FRα on a small subset of embryonic cells may similarly be critical to capture folates from maternal circulation and deliver them into the growing fetus (9). However, the function of FRα on the apical surfaces of other epithelial cells (e.g. uterus, mammary ducts, fallopian tubes, alveolar epithelial cells, etc.) remains enigmatic, since their apical surfaces face an externally exposed lumen where folate concentrations are negligible. Moreover, the functions of the three other isoforms of FR (β, γ, and δ) cannot be explained by a need for folate uptake, since they are found on immune cells that have abundant folate transporters and are generally not rapidly proliferating (10–12). Thus, FRβ is expressed on monocytes and macrophages (13), FRγ is detected primarily on mature neutrophils (14), and FRδ seems to be restricted to regulatory T cells (12, 15). The functions of FRβ and FRδ are further confused by the fact that these receptors largely exist in nonfunctional states that do not bind folate (10, 12) (function of FRγ has not been examined yet), suggesting that folate transport cannot be their primary function.

In pondering possible alternative functions for FRβ, we noted that FRβ expression is enhanced, and its vitamin binding function is activated by immunosuppressive cytokines, spent cancer cell culture medium, and ascites fluid from cancer patients (10). We also observed that FRβ-expressing tumor associated macrophages (TAMs), and myeloid derived suppressor cells (MDSCs) are the only myeloid cells capable of regulating T cells under hypoxic conditions (16). However, it remained unclear whether FRβ was simply a marker of immunosuppressive myeloid cells or perhaps a regulator of their suppressive activities. To explore this hypothesis, we generated FRβ knockout C57BL/6 mice and examined their immune functions at the organismal, cellular and molecular levels (9).

## Materials and methods

### Study design

The objective of this study was to determine whether folate receptor β (FRβ) performs an immune-related function besides folate transport in monocytes and macrophages. To test this hypothesis, we generated FRβ knockout (KO) C57BL/6 mice and compared their immunologic functions with those of wild type (WT) mice. We characterized the FRβ KO mice, analyzed immune-related gene and protein expression, evaluated responses to inflammatory stimuli, and examined tumor growth in both WT and KO mice.

Sample sizes were determined based on previous experience and pilot experiments to achieve sufficient statistical power. For animal studies, at least 3–7 mice per group were used, as indicated in the figure legends. Mice were randomly assigned to experimental groups. Investigators were not blinded during data collection and analysis. All animal studies were approved by the Purdue University Institutional Animal Care and Use Committee.

For human cancer patient survival analysis, publicly available data from The Cancer Genome Atlas (TCGA) were used. No statistical method was used to predetermine sample size for the human data analysis.

Experiments were performed at least three times independently unless otherwise noted. No data were excluded from the analyses.

### Cell lines

TRAMP C2 cells (Murine prostate adenocarcinoma) were purchased from the ATCC and cultured in DMEM medium supplemented with 10% FBS, 4 mM L-glutamine, 5 μg/mL insulin, 10 nM dehydroisoandrosterone and 1% penicillin/streptomycin. MC38 cells (murine colon adenocarcinoma) were purchased from Kerafast and cultured in DMEM medium with 10% FBS, 2mM L-glutamine, 0.1 mM nonessential amino acids, 1 mM sodium pyruvate, 10 mM HEPES, 50ug/mL gentamycin sulfate, and 1% penicillin/streptomycin. For all experiments, cells were used within 10 passages from purchased stocks.

### Animal husbandry

Six- to eight-week-old male or female wildtype C57BL/6 mice were purchased from Charles River. FRβ and FRδ knockout mouse breeders were generated by Richard Finnell at Baylor College of Medicine and bred at the Purdue University animal facility (9). Mice were housed in accordance with protocols approved by Purdue University Animal Care and Use Committee. Water, regular rodent diet (Envigo, #2018S/2018SC) or folate-deficient chow (Envigo, #TD.00434) were freely available. For all studies on tumor-bearing mice, mice were fed folate-deficient chow for two weeks before tumor implantation and maintained on the chow for the duration of the studies. Briefly, commercially available mouse chow contains megadoses of folic acid that raise the serum folate concentrations of the mice to ~700 nM. Physiological folate concentrations are however only 20 nM. Therefore, to reduce the folate levels in the mice to normal concentrations, we have maintained the mice on a low folate diet. These physiological folate concentrations are necessary to observe physiologic interactions of folate and folate-linked fluorescent dyes with endogenous folate receptors.

### Knockout mice

FRβ knockout mice were created by insertional mutagenesis in the Richard Finnell lab as described earlier (9). These mice were then backcrossed onto the C57BL/6J strain (Charles River) using inbred SWV background *Folr2* ^(+/-)^ heterozygous male mice (from the Finnell lab) with C57BL/6 females in the Amaya Puig-Kröger lab to generate the final FRβ knockout (*Folr2* ^-/-^) mice. FRδ knockout mice were generated in collaboration with Purdue University’s Transgenic and Genome Editing Facility using the purchased embryos (B6.129P2(Cg)-*Izumo1r^tm1.1Salb^/Mmnc*, Item# 037093-UNC-EMBRYO, MMRRC) (17). Tail tips (2-5mm) were collected from 2–3 weeks old FRβ KO pups in accordance with NIH Animal Research Advisory Committee (ARAC) Guidelines for Genotyping Mice & Rats. Samples were then processed as previously described (18). After PCR, samples were separated by electrophoresis on 2.5% agarose gels at 150 volts for 30 minutes, and DNA was imaged using a Blue View Transilluminator (Vernier Bio-Technology).

### Hematoxylin and eosin (H&E) staining of spleens

Whole spleens were harvested, fixed in formalin (10%), and H&E stained slides were prepared by following the standard protocol (19). For magnification control, hearts and livers were also stained with the spleens.

### Analysis of tumor growth and immune cell phenotype in WT, FRβ KO and FRδ KO mice

Male and female WT or FRβ KO or FRδ KO mice were inoculated subcutaneously with TRAMP-C2 cells (2×10^6^ cells/mouse in FRβ KO, 0.5×10^6^ cells/mouse in FRδ KO) or MC38 cells (4×10^5^ cells/mouse). Tumor sizes were measured 2–3 times per week and volumes were calculated as (L*W*W)/2, where L is the length, W is the width of the tumor. When tumors reached 1000-1500mm^3^, mice were sacrificed and tumors were dissociated with tumor dissociation kit (Miltenyi, Cat#130-096-730). Cells were filtered through a 70-μm cell strainer and treated with RBC lysis buffer (Biolegend, #420301) to deplete erythrocytes. After washing 2x with cold PBS, the resulting single cell suspensions were resuspended in FACS buffer, stained with Zombie Violet dye (BioLegend, Cat#423114), and incubated with anti-mouse TruStain FcX™ (BioLegend, Cat#101319) for 5–10 minutes on ice. Cells were then washed 2x with PBS and then stained with antibodies listed in **Supplementary Table S1**. For antibody staining, cells were incubated with antibodies for 30min on ice. For small molecule staining, cells were incubated with FA-Cy5 (10nM) in the presence or absence of FA-glucosamine (1uM) for 1h. After washing twice with cold PBS, cells were analyzed by flow cytometry for the desired phenotypic markers: Monocyte-like (M)-MDSCs were identified as CD45+CD11b+Ly6C+, while granulocyte-like (G)-MDSCs were identified as CD45+CD11b+Ly6G+. Tumor associated macrophages (TAMs) were identified as CD45+CD11b+Ly6G-Ly6C-F4/80+. Additional analysis of MDSCs and TAMs included staining with anti-PD-L1 antibody and folate-Cy5 in the presence or absence of folate-glucosamine (competition). T cells were identified as CD45+CD3+. CD69 was employed as T cell activation marker, while PD1 was used as T cell suppression marker. All flow cytometry analyses were conducted on an Attune™ NxT Acoustic Focusing Cytometer (Invitrogen). Data were analyzed with Attune™ NxT Software.

Differentiation and polarization of murine bone marrow derived macrophages and bone marrow derived MDSCs.

Murine bone marrow cells were isolated from tibias and femurs of WT or FRβ KO male C57BL/6 mice as previously described (20). Cells were differentiated to BMDMs by culturing for 7 days in complete growth medium (regular or folate deficient RPMI 1640 medium supplemented with 10% FBS and 1% Penicillin/streptomycin) containing 20 ng/mL recombinant mouse M-CSF (Biolegend). The resulting macrophages were polarized into M1-like macrophages by treatment for 24 hours in complete growth medium containing recombinant mouse IFNγ (20 ng/mL; Biolegend) plus 1 ng/mL lipopolysaccharide (Biolegend), or M2-like macrophages by incubating for 48 hours with 20ng/mL IL-4 and 20ng/mL IL-13. IL-6 was analyzed in cell culture supernatants by ELISA and cells were harvested for RNA sequencing.

BM-MDSCs were obtained by culturing the isolated bone marrow cells for 7 days in complete growth medium containing 40ng/mL recombinant mouse GM-CSF (Biolegend) and 40ng/mL IL-6 (Biolegend). Mouse IL-6 and IL-10 in cell culture supernatants were analyzed using mouse IL-6 (Biolegend, # 431304) and IL-10 (Biolegend, # 431414) ELISA kits, respectively, according to the manuals.

### Analysis of fibrosis and macrophage phenotypes in a bleomycin induced pulmonary fibrosis model

Eight-week-old WT or FRβ KO male mice were instilled with 0.75mg/kg bleomycin, as previously described (20), and body weights were monitored throughout the study. Mice were imaged by micro-CT on days 14 and 21 post bleomycin instillation as described previously (21) to quantitate lung fibrosis. Bronchoalveolar lavage (BAL) fluids were harvested on day 21 and pelleted the cells by centrifugation for qPCR analysis of macrophage phenotypic markers.

### qPCR analysis of RNA expression

For RNA isolation from mouse tissues, liver, kidney, and lung samples were freshly collected following euthanasia and homogenized in cold DNA/RNA Shield buffer (Zymo Research, #R1100-50). RNA was then extracted using the Direct-zol RNA Miniprep Kit (Zymo Research, #R2051), according to the manufacturer’s instructions.

For RNA isolation from mouse macrophages, the cells were lysed in cold DNA/RNA Shield buffer (Zymo Research, #R1100-50) followed by RNA extraction using the Quick-RNA™ MicroPrep kit (Zymo Research, #R1051) and reverse-transcribed to cDNA using High-Capacity cDNA Reverse Transcription Kit (Applied Biosystems™, #4374966) according to the manufacturer’s instructions as previously described (20). qPCR was performed using the iTaq™ Universal SYBR Green SuperMix (Bio-Rad Laboratories, #1725121), iCycler thermocycler, and iCycleriQ 3.0 software to examine the expression of folate transporters and M2-macrophages markers. Primer sequences for qPCR are shown in **Supplementary Table S2**.

### RNA-seq of BMDMs

Total RNA was extracted from three mice per group, purified from mouse BMDM cells, pooled in each group, and sent to Novogene for QC and RNAseq analyses (Novoseq PE150). RNA samples were sequenced using an Illumina sequencer using a paired-end protocol, targeted read-length of 150 bp, and >40 million total reads per sample. Data quality control (adapter trimming and removal of reads when bases with quality scores <5 constituted >50% of the reads) was performed at Novogene. Reads were aligned to mouse (mm10) reference genome using HISAT2 aligner (22). The reads mapped to each gene were calculated at Novogene. The EdgeR Bioconductor package (23) was used to perform the differential expression analysis. Genes with cutoffs (pvalue < 0.05 and |log2FoldChange| > 1) were denoted as Differentially Expressed Genes (DEGs). Heatmaps and bar plots were created using the R-package pheatmap (24) and ggplot2 (25), respectively. GO enrichment analysis was performed by Novogene.

### T cell suppression assay

Macrophages were obtained by harvesting bone marrow from healthy WT and FRβ KO C57BL/6 female mice followed by differentiation in the presence of mouse M-CSF (20 ng/mL) for 7 days. Cells were then polarized to M2-like macrophages by culturing 48 hours in IL-4 (20 ng/mL) and IL-13 (20 ng/mL) containing media (folate deficient RPMI 1640 medium supplemented with 10% FBS and 1% Penicillin/Streptomycin). T cells were obtained separately starting with harvesting spleen from healthy female WT C57BL/6 mice followed by dissociation into single cell suspension through triturating, filtration with a 70μm cell strainer, and washing the strainer with 2% FBS/PBS containing 1mM EDTA. Next, RBCs in the splenocytes were lysed using RBC Lysis Buffer (BioLegend, #420302) and T cells were isolated using Dynabeads™ Untouched™ Mouse T Cells Kit (Invitrogen™, #11413D) followed by activation in IL-2 (50 U/mL) and CD3/CD28 beads (2:1 bead-to-cell ratio) for 48 hours. The T cells were then added to the above M2-macrophages and incubated for 18 hours under hypoxic conditions in presence of IL-2, IL-4, IL-13, and CD3/CD28 beads as described above and in the presence or absence of 5 μg/mL mouse anti-FRβ antibody (26). Finally, the cells were stained as described in the “Analysis of tumor growth and immune cell phenotype in WT and FRβ KO mice” section and the % of CD69+ T cells was quantitated as a measure of T cell activation. The % suppression was calculated as (1 - activation with macrophages/activation without macrophages)×100.

### Survival analyses for human cancers

TCGA PanCancer RNA-seq datasets for Lung squamous cell carcinoma and kidney renal clear cell carcinoma were explored in cBioPortal (27). Then RNA-seq z-score plots were generated for FOLR2 gene, performed a quartile comparison of all the patients and plotted survival curves for FOLR2 high versus low patients. The z-scores -0.064-0.08 and 0.7-2.42 were considered as FOLR2 low and high, respectively for Lung squamous cell carcinoma and z-scores -0.56-0.09 and 0.66-2.53 were considered as FOLR2 low and high, respectively for kidney renal clear cell carcinoma.

### Immunofluorescent imaging of reduced folate carrier 1 and proton coupled folate transporter

Macrophages were obtained from healthy WT and FR-β KO C57BL/6 mice, seeded at 50,000 cells/well in 8-well chamber slides, and polarized into M2-like macrophages, as described above. After fixation, permeabilization, and blocking as described above, cells were treated with primary anti-RFC1 (reduced folate carrier 1, abcam, Catalog # ab193559, 1/1000 dilution) or anti-PCFT (proton coupled folate transporter, abcam, Catalog # ab25134, 10 µg/mL) antibody at 4°C overnight. The cells were then stained with AF488 conjugated Goat anti-Rabbit IgG secondary antibody (10µg/mL) and Hoechst 33342 nuclear dye (1µg/mL) and then imaged using Nikon A1R-MP multiphoton confocal microscope as described above.

### Flow cytometry analyses of reduced folate carrier 1 and proton coupled folate transporter

Macrophages were obtained from healthy WT and FR-β KO C57BL/6 mice, and polarized into M2-like macrophages, as described above. The cells were washed with PBS and stained with Zombie Violet dye and FcX as described in the “Analysis of tumor growth and immune cell phenotype in WT and FRβ KO mice” section. Then the cells were treated with primary anti-RFC1 (reduced folate carrier 1, abcam, Catalog # ab193559, 1/1000 dilution) or anti-PCFT (proton coupled folate transporter, abcam, Catalog # ab25134, 5 µg/mL) antibody along with the other surface antibodies (Anti-mouse CD45, CD11b, and F4/80) at 4 °C for 1 hour in dark. The cells were then washed and stained with AF488 conjugated Goat anti-Rabbit IgG secondary antibody (10µg/mL) for 1 hour at 4°C in dark. Followed by washing the cells, flow cytometry analyses were performed as described above. (26, 28, 29)].

### Statistical analysis

Statistical analyses were performed using GraphPad Prism software (V10). Data are presented as mean ± standard error of the mean (SEM). Unpaired two-tailed Student’s t-test was used to compare means between two groups. One-way ANOVA with appropriate *post-hoc* test was used to compare means among three or more groups. Two-way ANOVA was used to compare means with two independent variables. Log-rank (Mantel-Cox) test was used to compare survival curves. P values less than 0.05 were considered statistically significant. The specific statistical tests used for each experiment are indicated in the figure legends. No statistical methods were used to predetermine sample sizes.

## Results

### Characterization of FRβ knockout mice

**Figure 1:**
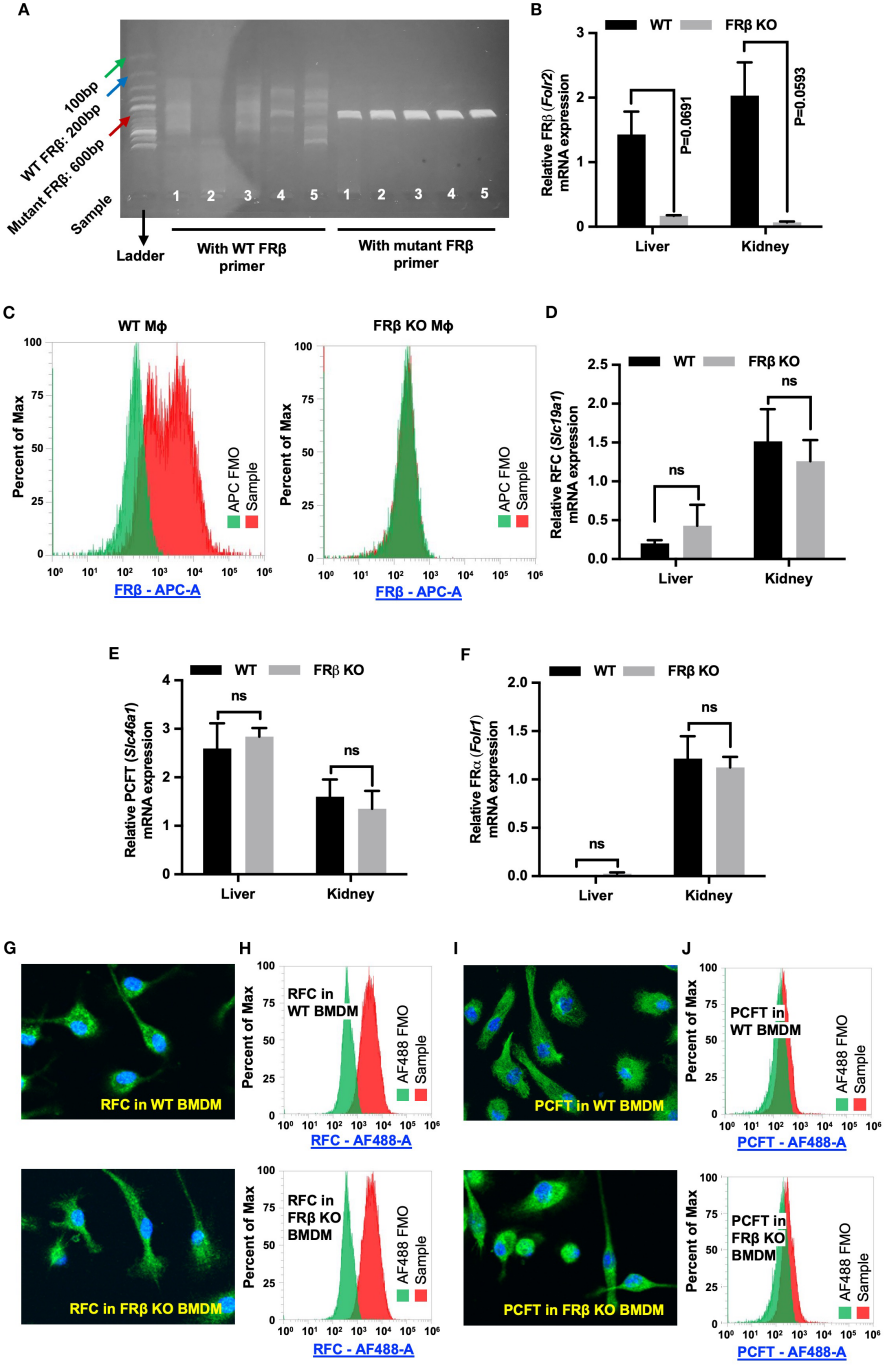
FRβ but no other folate transporters is absent from FRβ KO mice. **(A)** Tail clips were harvested from FRβ KO C57BL/6 mice for genotyping as described in the Methods. DNA from KO mice was amplified using either WT or mutant FRβ primers and amplicons were analyzed by DNA gel electrophoresis (n=5). Since the KO was generated by insertion of a neomycin resistance gene (NeoR) into the *Folr2* (FRβ) gene, the WT forward (FWD) primer was designed to pair with a segment of *Folr2* gene, while the mutant FWD primer paired with the *NeoR* gene. Both WT and mutant primer pairs used the same reverse primer, which targeted another segment of *Folr2* gene. The WT primers did not amplify the 200bp segment of *Folr2* gene demonstrating the absence of intact WT *Folr2* gene in the FRβ KO mice. The mutant FRβ primer amplified the anticipated 600bp segment of the mutated gene in the FRβ KO mice. **(C)** FRβ is detected in WT but not FRβ KO macrophages by flow cytometry. **(B, D–F)** FRβ mRNA is nearly absent in FRβ KO liver and kidney **(B)**, while RFC **(D)**, PCFT **(E)**, and FRα **(F)** transcript levels are unchanged between FRβ KO and WT tissues (n=3, normalized to β-actin). **(G–J)** RFC and PCFT protein expression and localization in bone marrow-derived macrophages are similar in WT and FRβ KO mice by confocal imaging and flow cytometry. All data are presented as mean ± SEM **(B, D-F)**. Unpaired T-test was used to compare the means between WT and FRβ KO mRNA levels **(B, D-F)**. ns, nonsignificant; *, P<0.05; **, P<0.01; ***, P<0.001; ****, P<0.0001. BMDM, Bone marrow derived macrophage. FMO, Fluorescence minus one. Mɸ, Macrophages.

**Figure 2:**
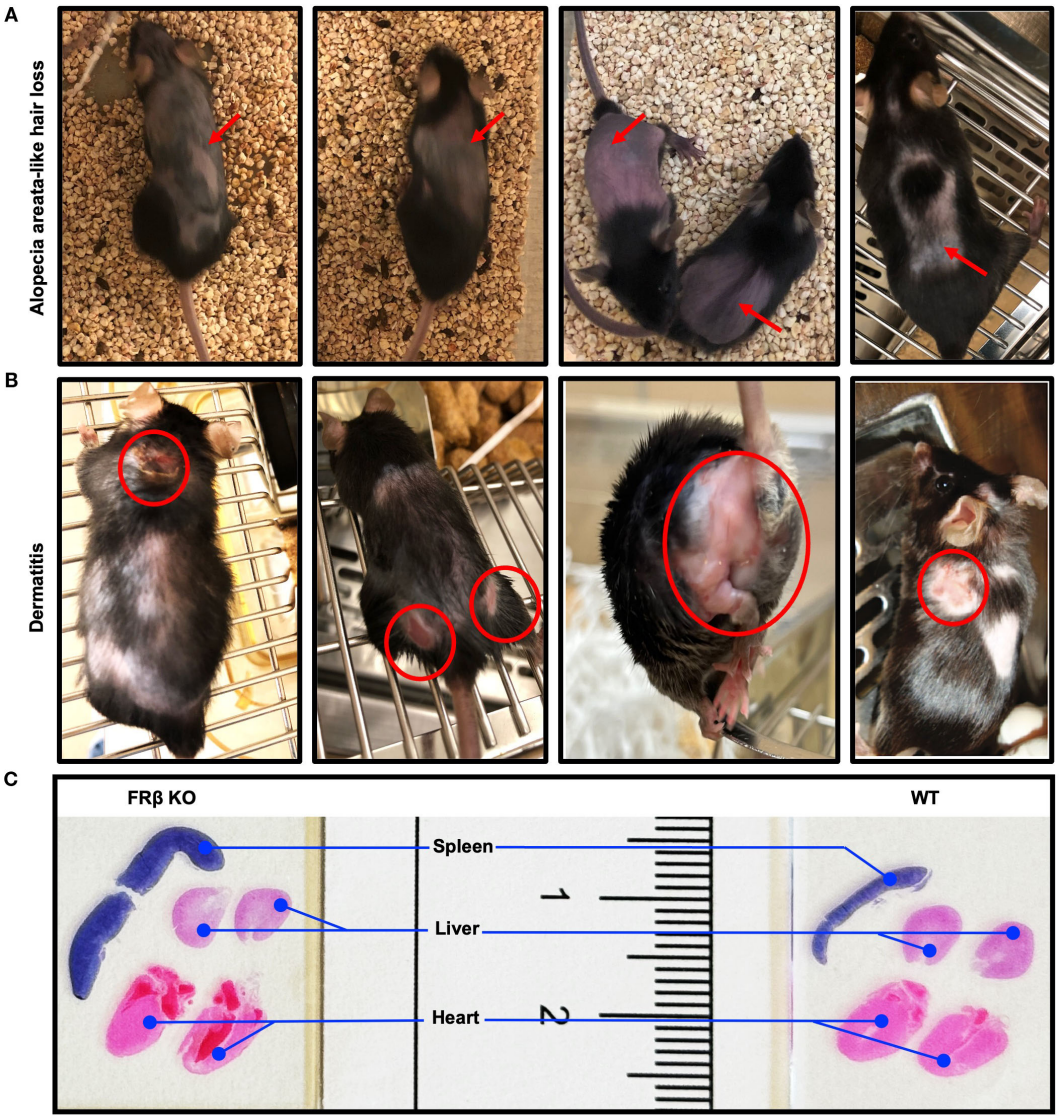
FRβ KO mice developed spontaneous autoimmune disease-like symptoms. **(A)** Images of FRβ KO C57BL/6 mice showing areas of spontaneous alopecia areata-like hair loss. **(B)** Images of FRβ KO C57BL/6 mice showing areas with spontaneous dermatitis. **(C)** Hematoxylin and eosin (H&E) stained spleen, liver and heart sections from FRβ KO and WT C57BL/6 mice showing larger spleen in FRβ KO mouse.

FOLR2 is a myeloid-specific gene for folate receptor beta (FRβ) that is expressed on a few subsets of monocytes and macrophages, but absent from essentially all other cells of the body (30–34). Because most copies of FRβ on myeloid cells in healthy individuals do not bind folic acid (**Supplementary Figure S1**) (10), and since the majority of FRβ on MDSCs and TAMs in tumor tissues is also nonfunctional, we hypothesized that FRβ might perform a function unrelated to folate transport, perhaps one involving immune regulation. To test this hypothesis, we generated an FRβ knockout (KO) mouse and compared its immunologic functions with those of wild type (WT) mice (9). As shown in **Figures 1A-C** and **Supplementary Figures S2, Figures S3**, insertional mutagenesis of the FRβ (*Folr2*) gene yielded mice in which the WT FRβ could not be detected, but in which FRα and both major folate transporters (RFC and PCFT) were transcribed (**Figures 1D-F**) and expressed (**Figures 1G–J**, **Supplementary Figure S3**) at normal levels. Because RFC and PCFT are known to supply most, if not all, folate needs of mature animals (5), it was not surprising that FRβ knockout mice were viable, fertile, and capable of growing to normal size. In fact, it was not until the KO mice were monitored for longer periods of time that it was observed that a substantial fraction (~20%) displayed spontaneous hair loss (**Figure 2A**), enlarged spleens (**Figure 2C**), and an unresolved dermatitis (**Figure 2B**) that were generally more severe in females than males and characterized by an elevated accumulation of macrophages in the affected tissues (**Supplementary Figure S4**). Because these symptoms were independent of the folate content in the diet, and since the KO mice had normal levels of the folate transporters that mediate uptake of folate into essentially all cells of the body (3–5), we conclude that inadequate folate is not the cause of these abnormalities. Instead, since the same symptoms can be logically attributed to an immune dysregulation (35), we hypothesized that deletion of FRβ might somehow alter the immunosuppressive functions of the macrophages. The studies below are designed to test this hypothesis.

### Comparison of immune-related protein and gene expression between WT and FRβ KO mice

**Figure 3:**
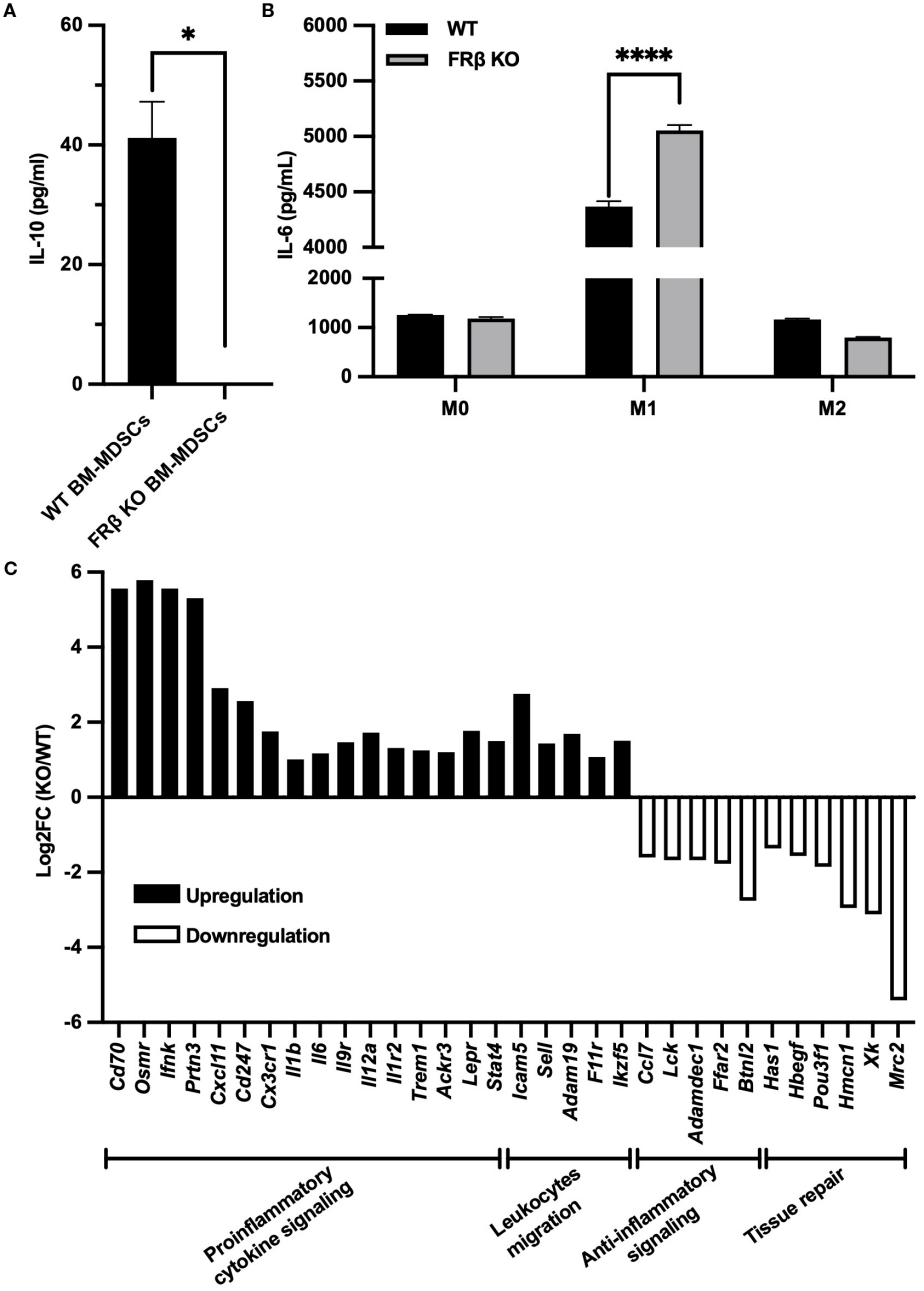
Immune-related gene expression in FRβ KO mice shows a shift towards a more inflammatory phenotype compared to WT mice. FRβ KO bone marrow-derived MDSCs secrete less IL-10 compared to WT mice **(A)**. M1-like macrophages from FRβ KO mice produce more IL-6 than WT mice **(B)**. RNA-seq of M1 macrophages (n=3 per group) shows gene expression changes in FRβ KO cells relative to WT, with upregulation of proinflammatory and chemotactic genes and downregulation of genes involved in anti-inflammatory signaling and tissue repair **(C)**. Unpaired T-test **(A, B)** was used to compare the means of different groups. ns, nonsignificant; *, P<0.05; **, P<0.01; ***, P<0.001; ****, P<0.0001.

To determine whether FRβ deletion might alter the immunologic properties of the FRβ KO macrophages, we collected bone marrow from both WT and KO mice and differentiated the myeloid cells into MDSCs or M0-, M1-, or M2-like macrophages (see Methods). As seen in **Figure 3A**, MDSCs from WT mice secreted high levels of IL-10, whereas IL-10 was undetectable in the culture medium from MDSCs of KO mice. In contrast, IL-6 was more highly produced by the KO mice than WT mice (**Figure 3B**). Since the KO macrophages appeared otherwise normal, and because IL-10 is an immunosuppressive cytokine whereas IL-6 is an immunostimulatory cytokine, the data suggest that there is a shift towards a more inflammatory phenotype in the KO mice (**Figures 3A, B**).

To obtain a more detailed assessment of the immune-related changes in gene expression that might be affected by deletion of FRβ, we next conducted RNA-seq analysis of bone marrow derived M1-like macrophages from KO and WT mice (**Supplementary Figures S5A, B**). As shown in **Figure 3C**, deletion of FRβ almost uniformly enhanced expression of pro-inflammatory genes (e.g. *Cd70, Osmr, Ifnk, Prtn3, Cxcl11, Cd247, Cx3cr1, Il1b, Il6, Il9r, Il12a, Il1r2*, etc.), while downregulating expression of both anti-inflammatory genes (*Ffar2, Btnl2, Adamdec1, Ccl7*, and *Lck*) and genes involved in tissue repair (*Has1, Hbegf, Pou3f1, Hmcn1, Xk, Mrc2*). This shift towards a more inflammatory phenotype was further evidenced by increases in the transcription of genes involved in leucocyte migration and motility, i.e. processes required for infiltration of immune cells into inflamed lesions.

**Figure 4:**
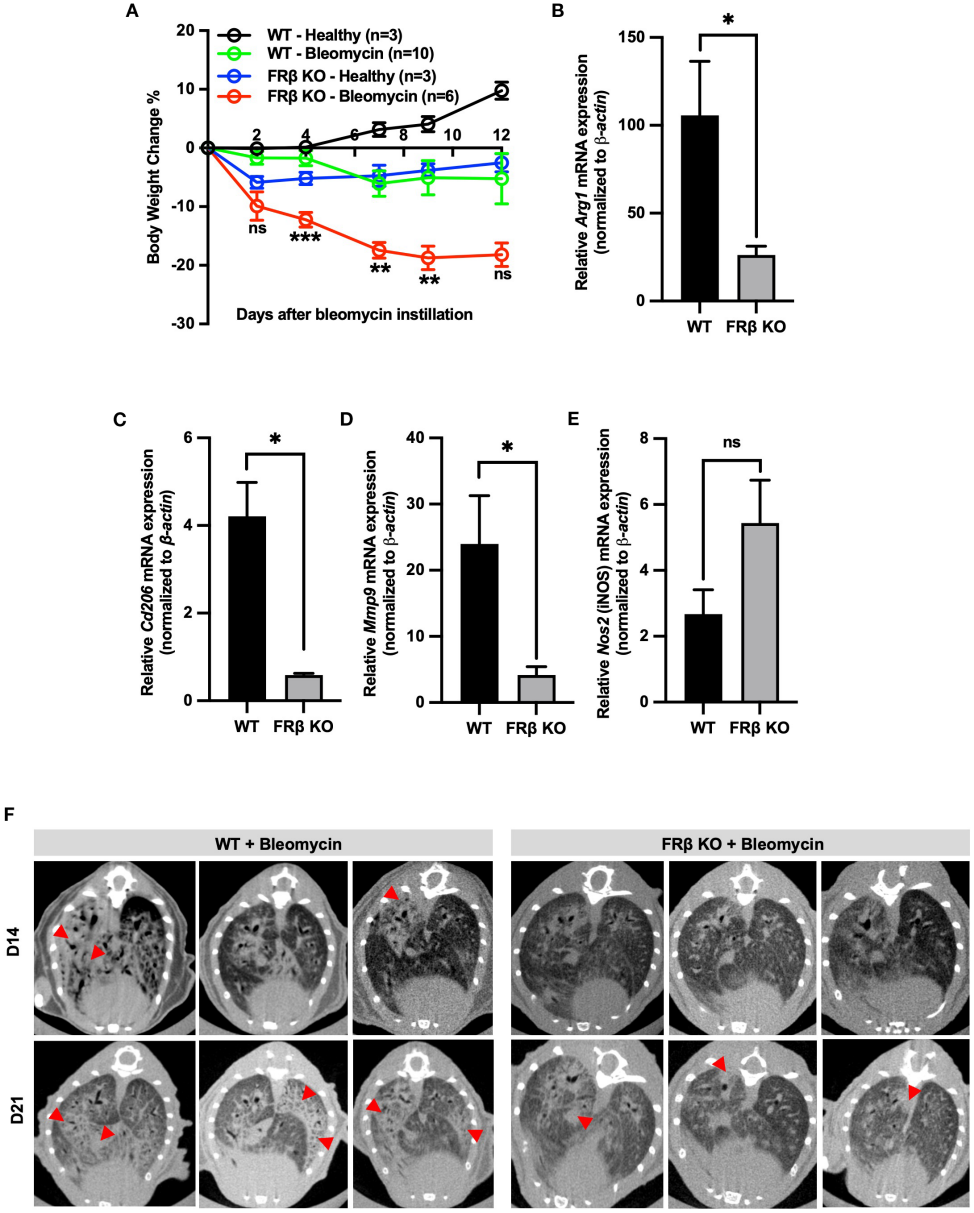
FRβ exerts an M2-like immunosuppressive effect in mice with bleomycin induced pulmonary fibrosis. Male WT or FRβ KO C57BL/6 mice were instilled with PBS (healthy control) or bleomycin to induce pulmonary inflammation and fibrosis. Bleomycin causes greater body weight loss in FRβ KO mice compared to WT **(A)**. On day 21, all mice were sacrificed and both bronchoalveolar lavage (BAL) fluid and lungs were collected for analysis. In BAL cells, FRβ KO mice (n=3 per group) show significantly reduced mRNA expression of M2-associated anti-inflammatory genes (*Arg1, Cd206, Mmp9*) **(B–D)** and a slight increase in mRNA for the M1 marker iNOS **(E)**. Micro-CT imaging reveals less pulmonary fibrosis in FRβ KO mice than WT controls (n=6 per group) **(F)**. All the data are presented as mean ± SEM **(A-E)**. Two-way ANOVA was used to compare the means among different groups and the statistics presented in **(A)** shows the differences between the WT-Bleomycin and KO-Bleomycin groups. Unpaired T-test was used to compare the means between WT and FRβ KO mRNA or protein levels **(B-E)**. ns, nonsignificant; *, P<0.05; **, P<0.01; ***, P<0.001; ****, P<0.0001.

Next, to determine whether this global repolarization towards a more inflammatory phenotype might affect the KO mouse’s response to an inflammatory stress, we induced inflammation in the lungs of both WT and KO mice by instillation of bleomycin and then compared their abilities to suppress the consequent emergence of inflammatory symptoms. As shown in **Figure 4A**, KO mice lost weight more rapidly following bleomycin administration, suggesting they were less capable of handling the inflammatory stress, i.e. consistent with their downregulation of anti-inflammatory markers (i.e., *Arg1, Cd206*, and *Mmp9*) (**Figures 4B-D**) and upregulation of a pro-inflammatory marker, iNOS (**Figure 4E**) in their bronchoalveolar lavage macrophages (CD45+F4/80+). Interestingly, the KO mice also exhibited less pulmonary fibrosis than the WT mice (**Figure 4F**), congruent with their diminished tissue repair capacity seen in the gene expression panel (**Figure 3C**). Taken together, these data demonstrate that FRβ plays a central role in regulating gene expression programs associated with macrophage polarization, and that its absence compromises macrophage-mediated immunosuppressive functions critical for controlling inflammation.

### Evaluation of the effect of FRβ deletion on tumor growth

**Figure 5:**
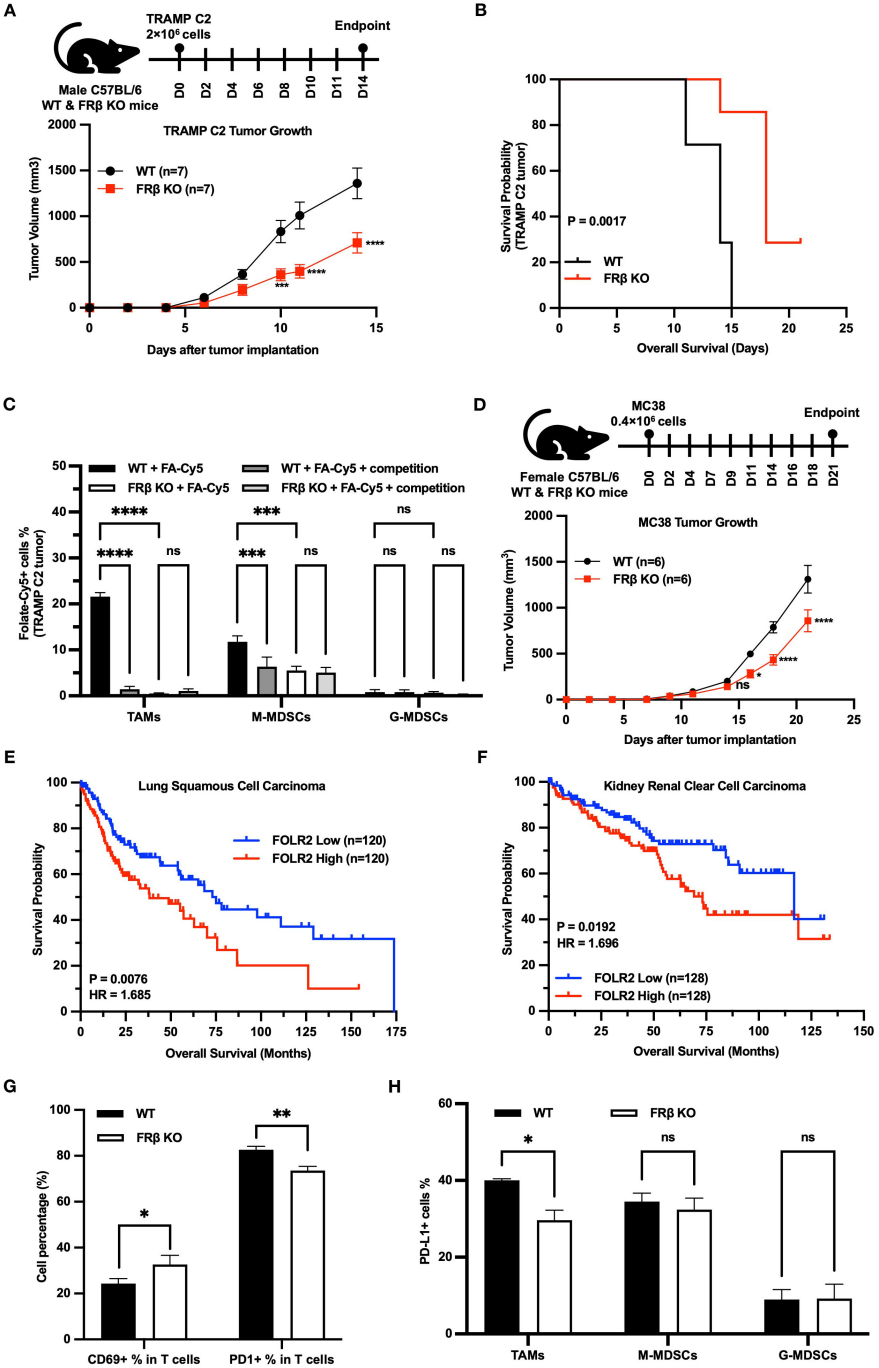
Loss of FRβ slows tumor growth, enhances anti-tumor immunity, and is linked to better survival in mice and humans. TRAMP C2 tumors grow significantly slower in FRβ KO mice (n=7 per group) **(A)**, resulting in improved survival compared to WT mice **(B)**. Myeloid cells (TAMs and M-MDSCs) from WT tumors bind the folate conjugate (FA-Cy5) significantly, while only background levels of FA-Cy5 binding are observed in cells from FRβ KO tumors **(C)**. MC38 colon tumors also grow slower in FRβ KO mice (n=6 per group) **(D)**. Analysis of TCGA datasets shows that high *FOLR2* (FRβ) expression correlates with reduced survival in patients with lung squamous cell carcinoma **(E)** and kidney renal clear cell carcinoma **(F)**. In TRAMP C2 tumors, FRβ KO mice have a higher percentage of activated (CD69+) T cells, lower PD-1 on T cells, and reduced PD-L1 on TAMs compared to WT **(G, H)**. These results indicate that FRβ promotes immune suppression and tumor growth in the tumor microenvironment.All data are presented as mean ± SEM **(A, C, D, G, H)**. Unpaired T-test **(G, H)** and 2-way ANOVA **(A, C, D)** were used to compare the means of different groups. Log-rank (Mantel-Cox) test was used to compare the survival probability between the two groups **(B, E, F)**. ns, nonsignificant; *, P<0.05; **, P<0.01; ***, P<0.001; ****, P<0.0001.

Because of the relevance of immune function to tumor growth (36), we next investigated whether syngeneic tumors might grow at different rates in KO and WT mice. As shown in **Figure 5A**, TRAMP C2 prostate tumors grew more slowly in KO than WT C57BL/6 mice, resulting in an overall survival that was predictably better in the KO than WT mice (**Figure 5B**). Although total white cells (CD45+), myeloid cells (CD45+CD11b+), macrophages (CD45+F4/80+), Tregs (CD45+FoxP3CD4+CD25+) and T cells (CD45+CD3+) did not differ between WT and KO tumors (**Supplementary Figure S6A**), the myeloid cells were FRβ+ in the WT mice but FRβ- in the KO mice was then confirmed by flow cytometry (FA-Cy5) (**Figure 5C**). Moreover, to establish that this inhibition of tumor growth was not an aberrant property of the TRAMP C2 tumor model, we repeated the study in C57BL/6 mice implanted with MC38 colon cancer cells. As shown in **Figure 5D**, MC38 tumors also grew slower in KO than WT mice. Taken together, these data demonstrate that FRβ is critical to the immunosuppressive function of TAMs and MDSCs.

Next, to determine whether a similar dependence of tumor growth on FRβ expression might also occur in humans, we used the TCGA database to examine whether a correlation might exist between FRβ expression and the probability of survival in human cancer patients. As shown in panels E & F, a negative relationship does indeed exist between FRβ expression and patient survival, arguing that the murine data have relevance to human immuno-oncology. Specifically, in both lung squamous cell carcinoma (**Figure 5E**) and kidney renal clear cell carcinoma (**Figure 5F**), patients with high FOLR2 (FRβ) expression had significantly shorter overall survival compared to those with low expression. For lung squamous cell carcinoma, the Log-rank P value was 0.0076 and the hazard ratio (HR) was 1.685, indicating a 68.5% higher risk of death in the high FOLR2 group. In kidney renal clear cell carcinoma, the Log-rank P value was 0.0192 and the HR was 1.696, again demonstrating significantly worse survival in the high FOLR2 group. These results support the conclusion that elevated FRβ expression correlates with poor prognosis and reinforce the clinical relevance of the findings in our murine models.

Next, to obtain a qualitative indication of the mechanism by which FRβ might influence the tumor microenvironment, we dissociated cells from TRAMP C2 tumors and examined the activation markers of their component T cells by flow cytometry. As shown in **Figure 5G**, CD69+ T cells were elevated in tumors from the KO mice, suggesting that T cell activation was increased in the absence of FRβ. Congruent with this observation, KO T cells also expressed lower levels of the checkpoint receptor PD-1, and TAMs from the same tumors displayed lower amounts of PD-L1(**Figure 5H**, **Supplementary Figure S6**).

**Figure 6:**
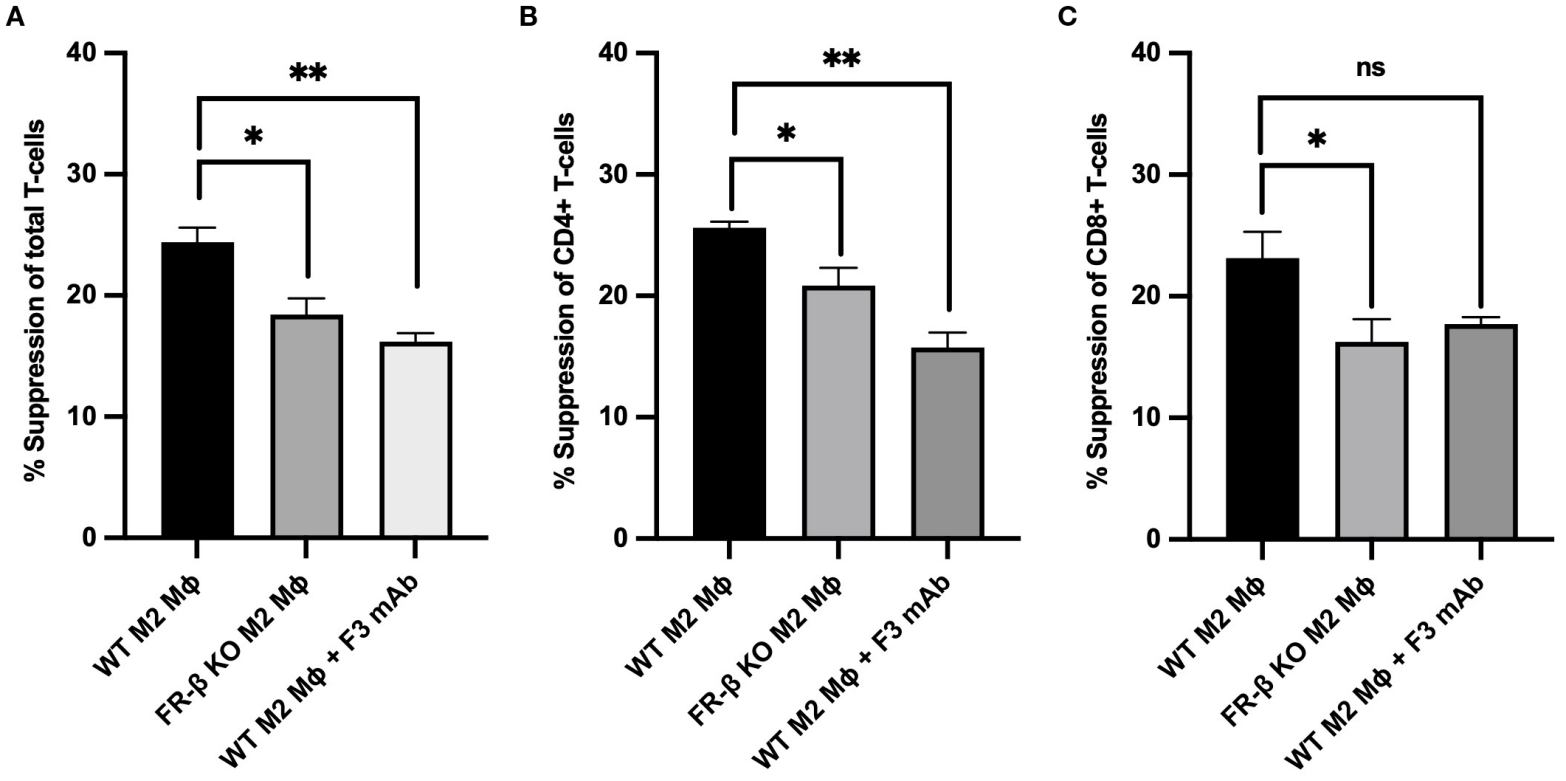
Absence or blockade of FRβ impairs the ability of M2-like macrophages to inhibit T cell activation. M2-like macrophages from FRβ KO mice were significantly less effective at suppressing T cell activation (measured by CD69 expression) in total T cells **(A)**, CD4+ T cells **(B)**, and CD8+ T cells **(C)** compared to WT M2-like macrophages. Blockade of FRβ on WT macrophages with anti-FRβ monoclonal antibody (F3 mAb) similarly reduced their suppressive capacity to the level observed in FRβ KO macrophages, indicating that FRβ is required for maximal macrophage-mediated T cell suppression (n=3 per group). One-way ANOVA was used to compare the means of different groups. ns, nonsignificant; *, P<0.05; **, P<0.01; ***, P<0.001; ****, P<0.0001. Mɸ, Macrophages.

Next, to explore whether the above changes in T cell phenotype might be mechanistically linked to the aforementioned changes in myeloid cell function, we generated M2-like macrophages from WT and KO mouse bone marrows and compared their capacities to suppress T cell activation *in vitro*. As shown in **Figures 6A-C**, M2 macrophages from KO mice were less effective in inhibiting CD69 expression (a T cell activation marker) in CD8+, CD4+ and total T cell populations than macrophages from WT mice, i.e. confirming that FRβ impacts the ability of macrophages to regulate T cell activation. That this intercellular communication could involve physical contact between FRβ on the macrophage and a cognate receptor on the T cell was then demonstrated by showing that blockade of FRβ with an anti-FRβ monoclonal antibody reduced the suppressive function of WT macrophages to the level observed with KO macrophages (**Figures 6A-C**, **Supplementary Figure S7**).

**Figure 7:**
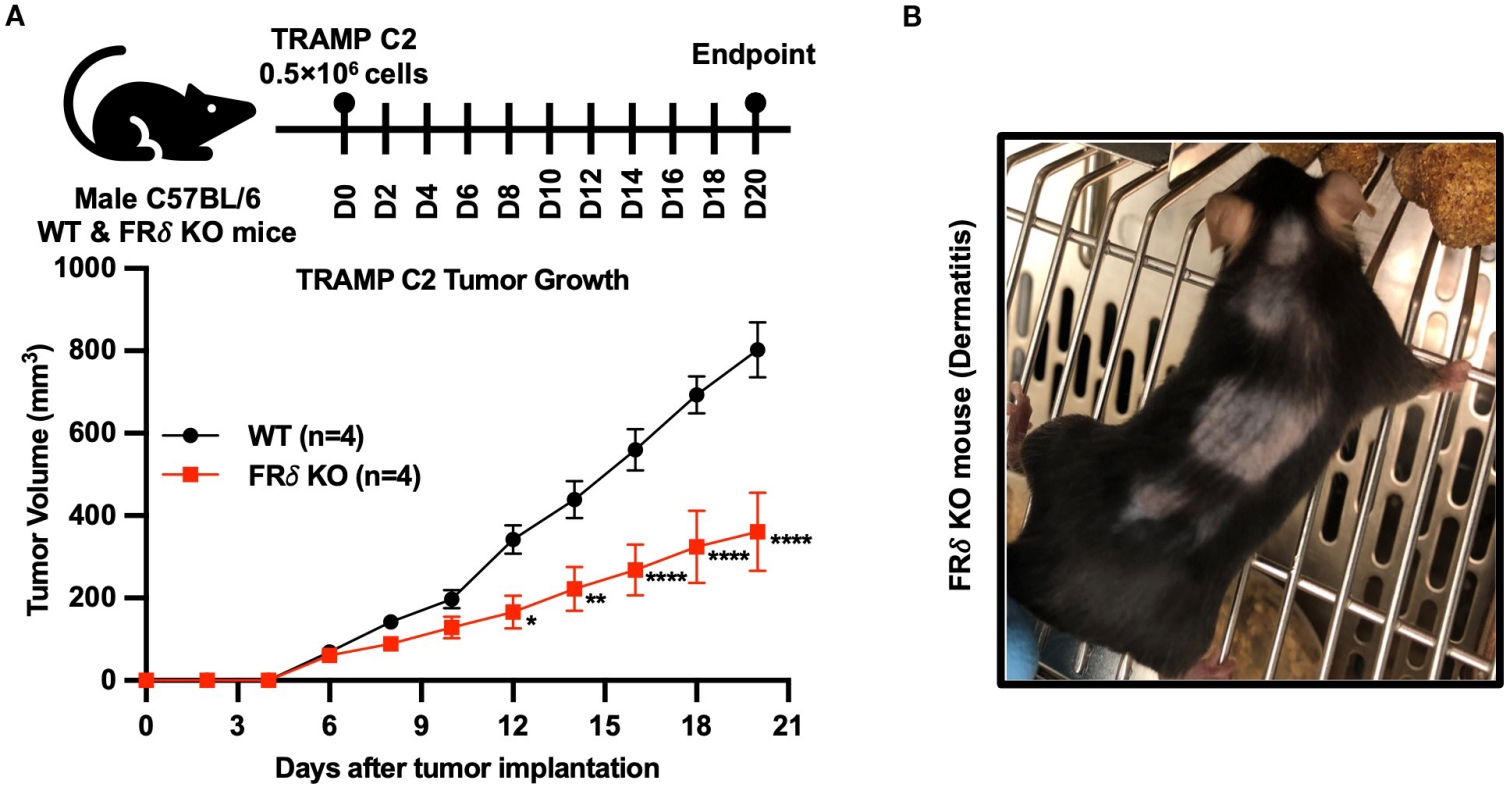
The absence of FRδ also reduces growth of TRAMP C2 tumors in C57BL/6 mice. TRAMP C2 tumors grew significantly slower in FRδ KO mice compared to WT controls (n=4 per group) **(A)**. In addition, some FRδ KO mice developed dermatitis, as illustrated in the representative image **(B)**. All of the data are presented as mean ± SEM **(A)**. 2-way ANOVA **(A)** was used to compare the means of WT or FRδ KO groups. ns, nonsignificant; *, P<0.05; **, P<0.01; ***, P<0.001; ****, P<0.0001.

Finally, noting that a different isoform of the folate receptor, namely folate receptor delta (FRδ), constitutes a marker for highly immunosuppressive regulatory T cells (Tregs) (37), and recognizing that most FRδ also does not bind folic acid (38), we reasoned that FRδ might similarly perform an immunosuppressive function in Tregs, i.e. much like FRβ in TAMs/MDSCs. To obtain an initial indication of whether this hypothesis might be correct, we generated FRδ knockout mice and compared the growth rates of Tramp C2 tumors in FRδ KO and wild type C57BL/6 mice. As shown in **Figure 7A**, Tramp C2 tumors indeed grew slower in FRδ KO mice. Moreover, as revealed in **Figure 7B**, some of these KO mice also developed dermatitis. Taken together, these data suggest that FRδ may also perform an immunoregulatory function.

## Discussion

Because folates are essential for cell proliferation, we undertook to determine why relatively quiescent cells such as TAMs and MDSCs express such high levels of a folate receptor (in addition to RFC and PCFT) and why this receptor is especially upregulated in immunosuppressive subsets of these cells (13, 16, 32). The studies performed here demonstrate that FRβ is required for the immunosuppressive properties of TAMs and MDSCs and that FRδ may be similarly essential for the suppressive functions of Tregs. Because concordant changes in cellular phenotype, gene and protein expression, response to antibody administration, and cytokine secretion have all been used to identify checkpoint receptors in other cells (39–43), we conclude that FRβ performs a checkpoint function in myeloid cells such as TAMs and MDSCs.

Although the mechanism by which FRβ mediates this immunosuppressive function was not fully resolved, a number of clues did emerge that place significant constraints on the nature of this mechanism. First, FRβ could not be detected in the nucleus of macrophages, either before or after their differentiation to an immunosuppressive M2-like state (see **Supplementary Figure S8**), suggesting that FRβ likely does not act as a transcription factor like other vitamin receptors (e.g. vitamin D receptor, vitamin A receptor, and FRα) (44–47). Second, the immunosuppressive activities of FRβ do not require folic acid, since folate deprivation had no effect on immunosuppression and because the reduced folate carrier and proton coupled folate transporter (i.e. the transporters required for folate uptake into virtually all cells of the body) were expressed at normal levels in the myeloid cells of the KO mice (**Figures 1D, E, GJ**). Third, several immunosuppressive functions of the FRβ-expressing TAMs/MDSCs did not require stimulation or activation by other immune cell types (except for the stimulation involved in differentiating the monocytes into M1 or M2 macrophages), since the dramatic differences in both gene expression and cytokine secretion were readily observed in isolated macrophages in culture (**Figures 3A, B**) and because the immunosuppressive functions of FRβ could be communicated to CD4+ and CD8+ T cells without involvement of other immune cell types (**Figures 6A-C**). In this respect, it was interesting to note that a monoclonal antibody to mouse FRβ could block suppression of T cell activation by FRβ+ macrophages, suggesting that physical contact between FRβ on the macrophage and a cognate receptor on the T cell might be involved. This possibility, in fact, is supported by the observation that FRδ on regulatory T cells mediates regulatory T cell immunosuppression of γδ-T cells via formation of a synapse between FRδ on the regulatory T cells and a protein termed Izumo-1 on the γδ-T cells (37).

The observation that expression of FRβ constitutes the only genetic difference between WT mice in which tumors grow rapidly and KO mice in which tumors grow slowly confirms that infiltrating myeloid cells have a significant impact on tumor growth (**Figures 5A, D**) (48). The specific upregulation of FRβ on the immunosuppressive subset of monocytic myeloid cells (32) further implies that methods that target drugs to FRβ (10, 49) should primarily concentrate the targeted drugs in the immunosuppressive subsets of TAMs and MDSCs, leaving the FRβ negative myeloid cells unperturbed. We have frequently used FRβ as a portal to deliver immune activators specifically into immunosuppressive TAMs and MDSCs, leading not only to their dramatic repolarization to inflammatory macrophages, but also to the consequent repolarization of most other immune cells in the tumor microenvironments (10, 16, 49). Thus, tail vein injection of a folate-linked TLR7 agonist into tumor-bearing mice has been shown to i) increase the proportion of monocyte-derived myeloid cells in the tumors, ii) increase the expression of inflammatory genes and decrease expression of anti-inflammatory genes in these infiltrating myeloid cells, iii) enhance infiltration of T cells, NK cells, dendritic cells and mast cells/basophils into the tumor microenvironment, iv) induce repolarization of these infiltrating T cells, dendritic cells and NK cells to a more pro-inflammatory phenotype, v) reduce tumor growth by ~50%, and vi) augment CAR T cell potency, all without directly engaging the cancer cells or perturbing the polarization of similar macrophages in healthy tissues (10, 16, 49). This observation here that deletion of FRβ not only alters gene expression in tumor infiltrating macrophages but also repolarizes cytotoxic T cells in the same malignant lesions is consistent with the hypotheses that repolarization of myeloid cells can shift the polarities of proximal lymphocytes in the same tumors (16, 36, 50). This observation is also consistent with the fact that retinoic acid, which induces the upregulation of FRβ on myeloid cells (51), exerts a general immunosuppressive effect on the entire immune system (52–55).

Although many mechanisms can be envisioned to explain the role of FRβ in immunosuppression, one mechanism that warrants further consideration emerges from the aforementioned analogy with FRδ (**Supplementary Figure S9**) (37). Thus, FRδ was originally identified on oocytes where it was assigned the name Juno and shown to function as the receptor for the spermatazoan protein, Izumo1 (38). As might be anticipated, docking of Izumo1 on the sperm with Juno on the egg results in engagement of the gametes and constitutes an essential step in fertilization (38). In looking for a possible receptor for FRδ on cytotoxic γδT cells, Zarin and colleagues (2023) discovered the expression of Izumo1 on T cells and demonstrated that Izumo1 on γδT cells served as the cognate receptor for FRδ on Tregs. The fact that FRβ is homologous to FRδ and that Izumo1 is also expressed on those T cells that interact with macrophages (37) raises the possibility FRβ might similarly serve as docking receptor for formation of an immunological synapse between macrophages and T cells. It will obviously be important to characterize the possible interactions of FRβ (on TAMs/MDSCs), FRγ (on neutrophils), and FRδ (on Tregs) with the different isoforms of Izumo on immune cells to determine whether the discovery of Zarin and colleagues (2023) might apply to other forms of folate receptors on other immune cells.

Finally, the observation that FRβ and PD-L1 both serve as checkpoint receptors on macrophages raises the question whether their immunosuppressive activities might differ. At least on the surface, both receptors are similarly upregulated on TAMs and MDSCs (16) and both receptors suppress T cell activation (16, 56). Moreover, knockout of both FRβ and PD-L1 shifts macrophage polarization towards a more inflammatory (M1-like) phenotype (57), and tumors in both KO mice grow much slower than in WT mice (58). If the aforementioned conjecture regarding FRβ serving as a receptor involved in T cell engagement is correct, then both FRβ and PD-L1 would also perform similar T cell docking functions. Thus, at least at the phenomenological level, FRβ may function similarly to PD-L1, however, more detailed investigations will be necessary to confirm or refine this hypothesis.

## Data Availability

All RNA-seq data are available in the NCBI database (Gene Expression Omnibus [GEO]) under the accession number GSE283674.

## Supplementary material

Supplementary Table 1 Antibodies and small molecule reagents for flow cytometry.

Supplementary Table 2 PCR primer sequences.
