# Supplementary material for: Folate receptor β performs an immune checkpoint function in activated macrophages
Source: Front Immunol. 2025 Sep 29;16:1638907. doi: 10.3389/fimmu.2025.1638907 (PMC12515634; doi:10.3389/fimmu.2025.1638907)
Supplement: Supplementary file 1 [file DataSheet1.docx]

**Supplementary Methods**

**Folate binding ability of FRβ on monocytes/macrophages**

Human peripheral blood mononuclear cells (PBMCs) were isolated from fresh peripheral blood using SepMate™-50 tubes (Stemcell) and cultured for 3 hours in monocyte attachment medium (PromoCell) at a density of 1 million/cm^2^, after which the cells were washed 4x with pre-warmed PBS and differentiated for 7 days (replenished medium on day 3) into unpolarized macrophages in folate deficient RPMI medium (10% FBS) containing 20 ng/mL of recombinant human macrophage colony stimulating factor (M-CSF) (Biolegend). The cells were then polarized to M2-like macrophages by incubating in folate deficient RPMI medium containing recombinant human IL-4 (20ng/mL, Biolegend) and IL-13 (20ng/mL, Biolegend) for 48 hours. The cells were analyzed on different days using an Attune™ NxT Acoustic Focusing Cytometer (Invitrogen). The percentage of FRβ+ and EC17+ cells were measured using a staining procedure similar to the one described in the “Analysis of tumor growth and immune cell phenotype in WT, FRβ KO and FRδ KO mice” section. However, an extra step was added after Zombie Violet staining, where the cells were stained with 100nM EC17 for 1 hour in the dark at room temperature. EC17, a folate-FITC conjugate, indicates the cells' ability to bind folate.

**Immunofluorescent imaging of mice skin**

Skin specimens were collected from FRβ KO and WT C57BL/6 mice post-euthanasia, fixed in 10% Neutral Buffered Formalin (StatLab Medical Products, #28600-2.5) and temporarily stored in 70% ethanol. The specimens were then embedded in paraffin and 4μm thin sections were generated using a microtome. Sections were then mounted on charged slides and dried in a 60°C oven. After drying, all slides were deparaffinized through 3 changes of xylene and rehydrated through graded ethanols to water in a Leica Autostainer XL.

Immunofluorescence: After deparaffinization, antigen retrieval was performed with EDTA pH 9.0 in a BioCare decloaking chamber at a temperature of 95C for 20 minutes. After cooling to 60C, slides were transferred to TRIS buffered saline (TBS) and marked with a hydrophobic pen before being placed on the BioCare intelliPATH automated stainer for the remainder of the procedure.

Slides were rinsed with TBS and then incubated in 2.5% normal goat serum for 20 minutes. Excess reagent was blown off and F4/80 1:100 (BioRad, MCA497R) was applied for 1 hour. Slides were rinsed twice in TBS and goat anti-rat 555 (Invitrogen, A21434) was applied to the tissue for 30 minutes. Slides were rinsed twice in TBS and then DAPI 1ug/mL (Invitrogen, 62248) was applied for 10 minutes. Slides were rinsed in TBS, removed from the intelliPATH stainer, placed into water and then cover slipped using Invitrogen ProLong Gold antifade reagent (P36934).

Digitization: A Leica Aperio VERSA 8 was used to take 20x images of the slides. Slides were scanned using 2 channels: blue, and orange. DAPI (blue), F4/80 (orange). These were then uploaded to Aperio eSlide manager.

**Localization assay of FRβ in human primary M2-macrophages**

Human monocytes were isolated and differentiated to M2-like macrophages as described in the Supplementary Methods section. The cells were seeded onto 8-well chamber slides (Thermo Scientific) at a density of 50,000 cells/well. Cells were washed with PBS (2x) and then treated with either folate-glucosamine (FA+, 200nM in 2% FBS in PBS) or the vehicle (FA-, 2% FBS in PBS) for 30 minutes at 37°C in a CO2 incubator. After washing (PBS, 2x), the cells were fixed in 4% paraformaldehyde (Fisher Scientific, # AAJ19943K2), washed (PBS, 3x), and permeabilized with 0.2% Triton X-100 (Alfa Aesar, #A16046). Then, after washing in 2x in PBS, the cells were blocked with 2% bovine serum albumin (BSA, Millipore Sigma, #A7030) and the respective wells were treated with the primary human anti-FRβ antibody(50) (m909-rabbit IgG antibody, 157µg/mL) at 4°C overnight. Cells were then washed (PBS, 3x) and treated with AF488 conjugated Goat anti-Rabbit IgG secondary antibody (Invitrogen, Catalog # A32731, 10µg/mL) for 1 hour at RT in dark. After washing again (PBS, 3x), the cells were stained with Hoechst 33342 nuclear dye (Thermo Scientific, Catalog # 62249, 1µg/mL) for 15 minutes at RT in dark, followed by washing (PBS, 3x). Cells were then submerged in 500 µL PBS and images were acquired using the 405nm (Hoechst 33342) and 488nm (AF488) lasers in Nikon A1R-MP multiphoton confocal microscope.

**Human FRβ and FRδ protein BLAST**

The human FRβ (Accession: P14207) and FRδ (Accession: A6ND01) protein sequences were downloaded from UniProt(52). Then a blastp was performed using NCBI “Align Sequences Protein BLAST” tool with default parameters(53) (BLOSUM62 matrix, RID: S20UW1WG114, Accession: Query_3982395).

**Supplementary Figures**

**A**

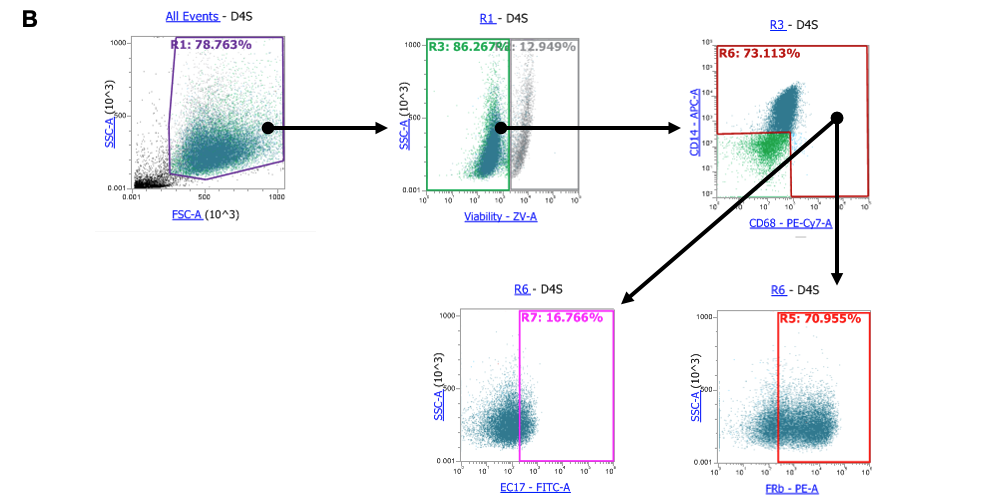


**Fig S1. Folate binding ability of FRβ on monocytes/macrophages.** Human monocytes were isolated from healthy donor blood as described in Supplementary Methods. The monocytes were cultured in presence of human M-CSF (20 ng/mL) for 7 days and then the cells were cultured in presence of human IL-4 (20 ng/mL) and IL-13 (20 ng/mL) for 2 days. EC17 (Folate-FITC) was used to determine folate binding capability of the monocytes/macrophages at different days through flow cytometry analyses (**A**). The CD14+ or CD68+ live cells were gated for assessing the percentage of cells expressing FRβ and the percentage of cells capable of binding EC17 (**B**, flow cytometry gating strategy). FMO, Fluorescence minus one.

**
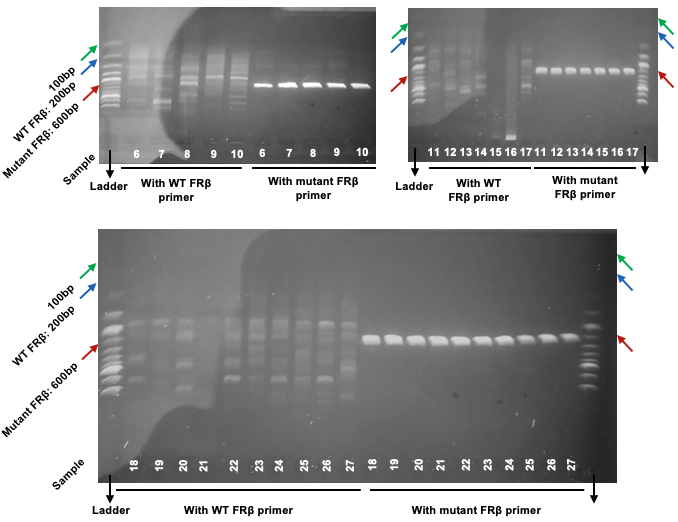
**

**Fig S2. Validation of the absence of FRβ expression by genotyping of FRβ KO mice.** Tail clips were harvested from FRβ KO C57BL/6 mice for genotyping as described in the Methods section. DNA from KO mice was amplified using either WT or mutant FRβ primers and analyzed by DNA gel electrophoresis (n=22). Note that the FRβ gene deletion was generated by insertional mutagenesis of a neomycin resistance gene (NeoR) into the Folr2 (FRβ) gene. The WT forward (FWD) primer was targeted to a segment of Folr2 gene, while the mutant FWD primer was targeted to the inserted NeoR gene. Both WT and mutant primer pairs used the same reverse primer which targeted another segment of Folr2 gene. The WT primers did not amplify the 200bp segment of the Folr2 gene, demonstrating the absence of the intact WT Folr2 gene in the FRβ KO mice. The mutant FRβ primer amplified the anticipated 600bp segment of the mutated gene in the FRβ KO mice.

**
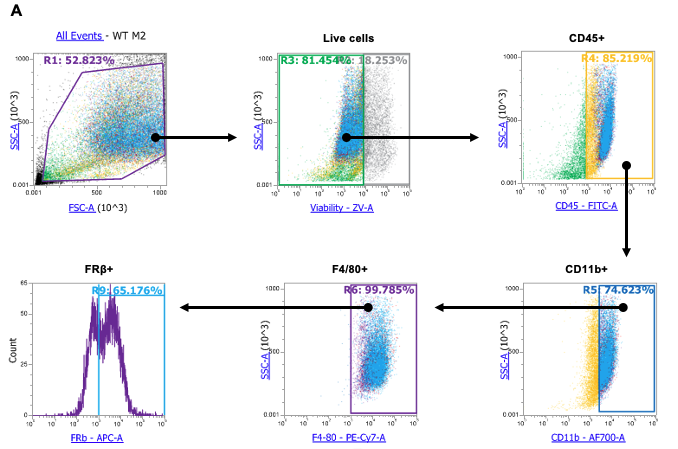
**

**
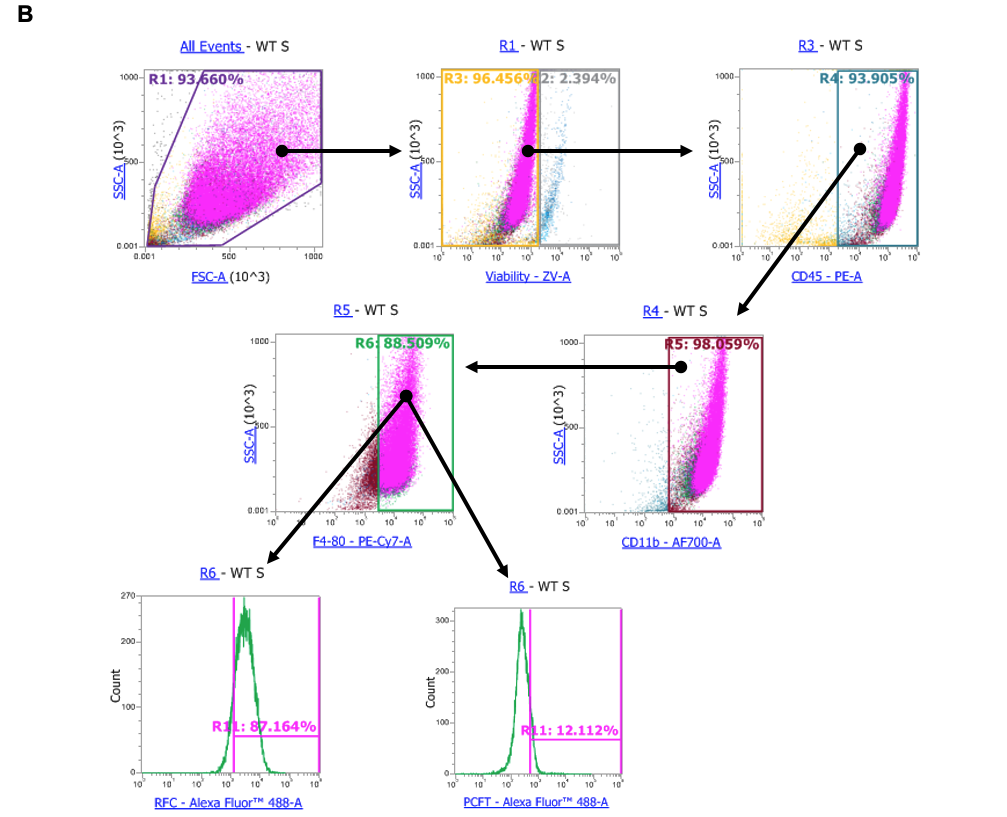
**

**Fig S3. Flow cytometry gating strategy for (A) FRβ, (B) reduced folate carrier (RFC), and proton coupled folate transporter (PCFT) levels in bone marrow derived macrophages from FRβ KO and WT C57BL/6 mice.** Bone marrow derived macrophages were obtained and differentiated to M2-like macrophages as described in Methods. The CD45+/CD11b+/F4-80+ macrophages were analyzed for FRβ+, RFC+, and PCFT+ cell quantification. The FRβ+, RFC+, and PCFT+ cell percentages were measured separately.


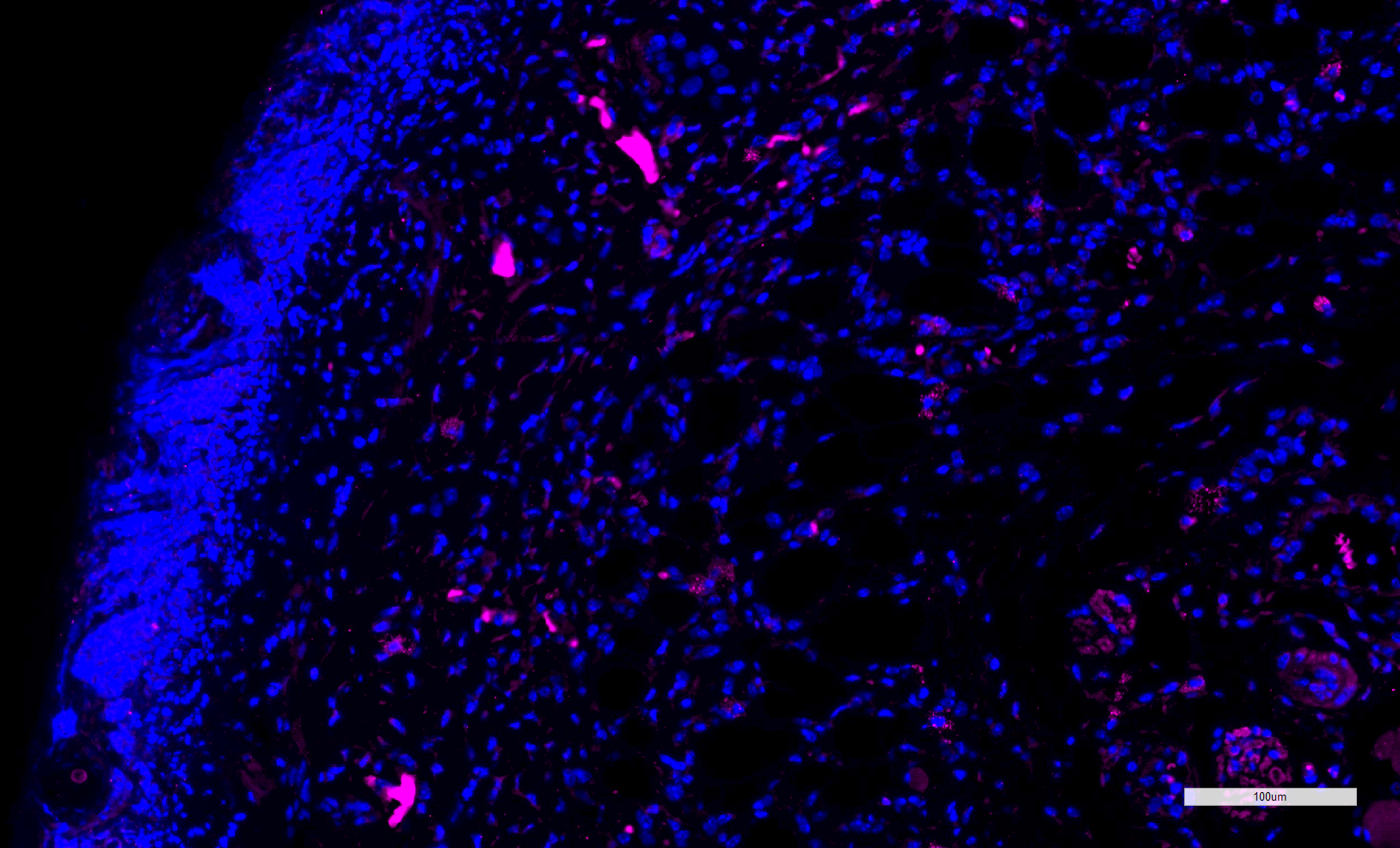

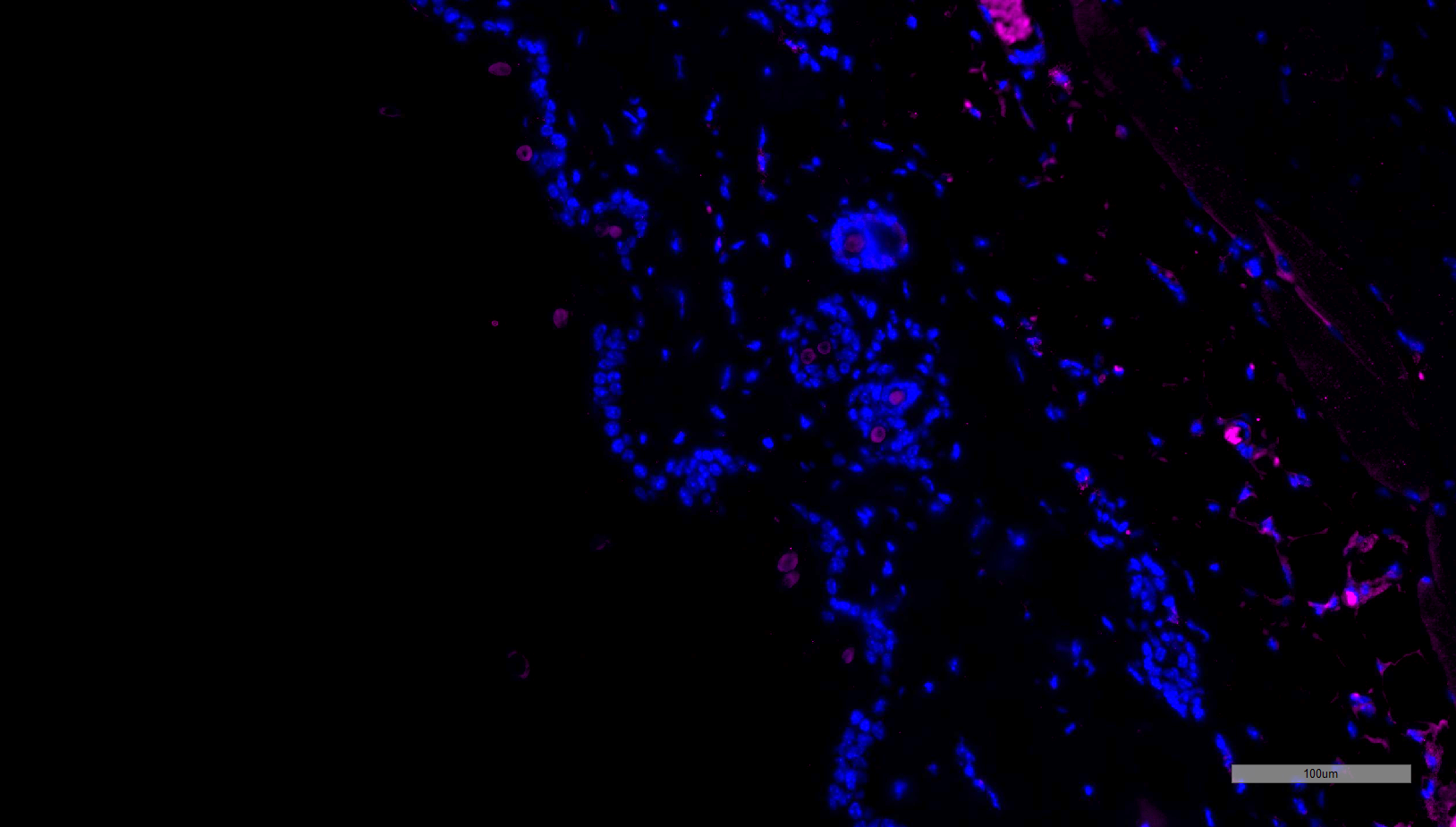

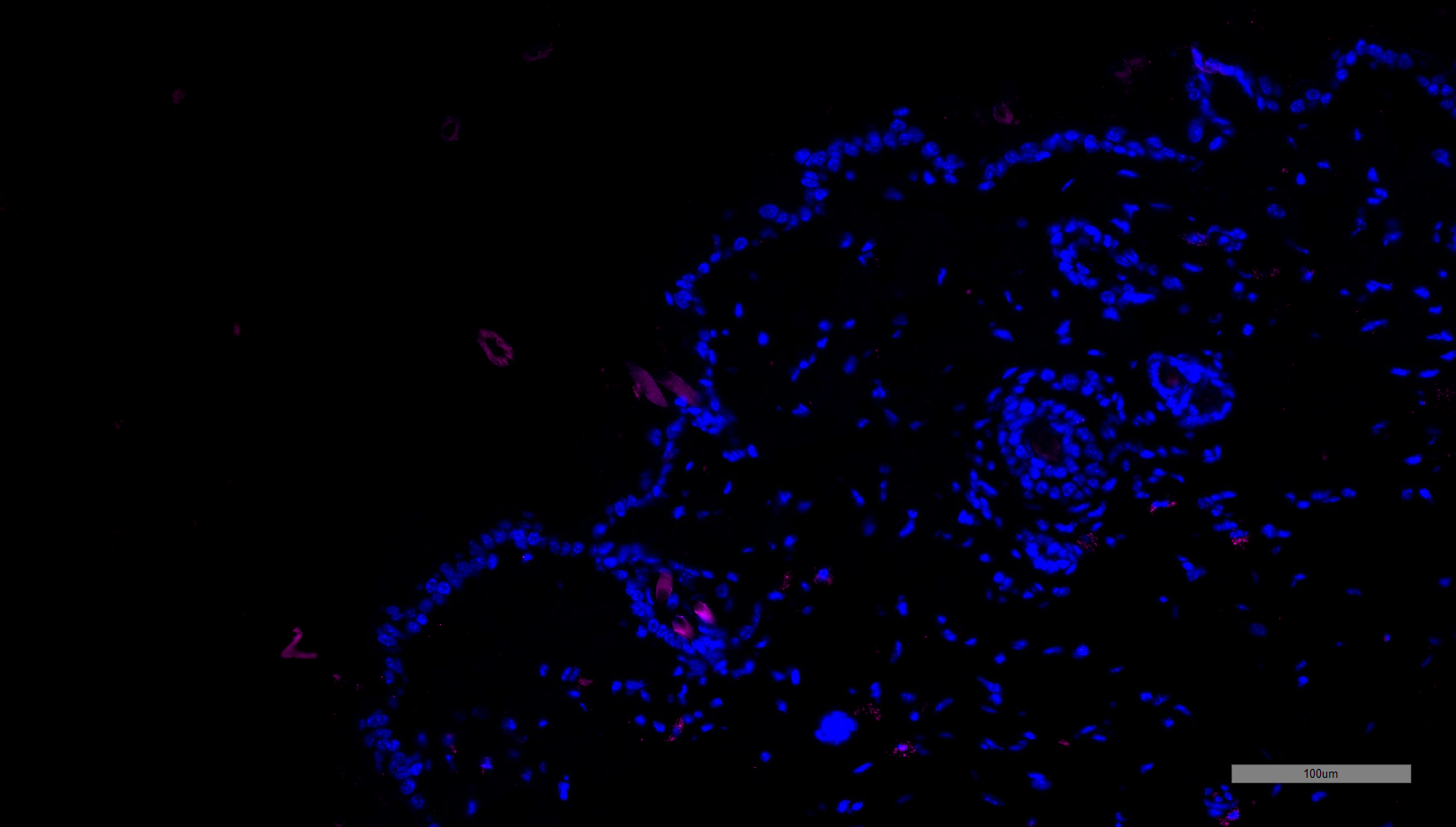


**Blue – DAPI | Pink – F4/80**

WT Healthy Skin

KO Dermatitis Skin

KO Healthy Skin

**Fig S4. Distribution of macrophages in the skin of FRβ KO and WT mice.** Skin specimens were collected from WT (left), KO healthy skin (middle) and KO dermatitis skin (right) mice post-euthanasia. After fixation and paraffin embedding, thin sections were mounted on slides followed by immunofluorescence staining with DAPI and F4/80 antibody. A higher number of macrophages (F4/80) were observed in the dermatitis affected FRβ KO mice skin, while the healthy skin specimen from WT C57BL/6 mice demonstrated the least number of macrophages (20x magnification).


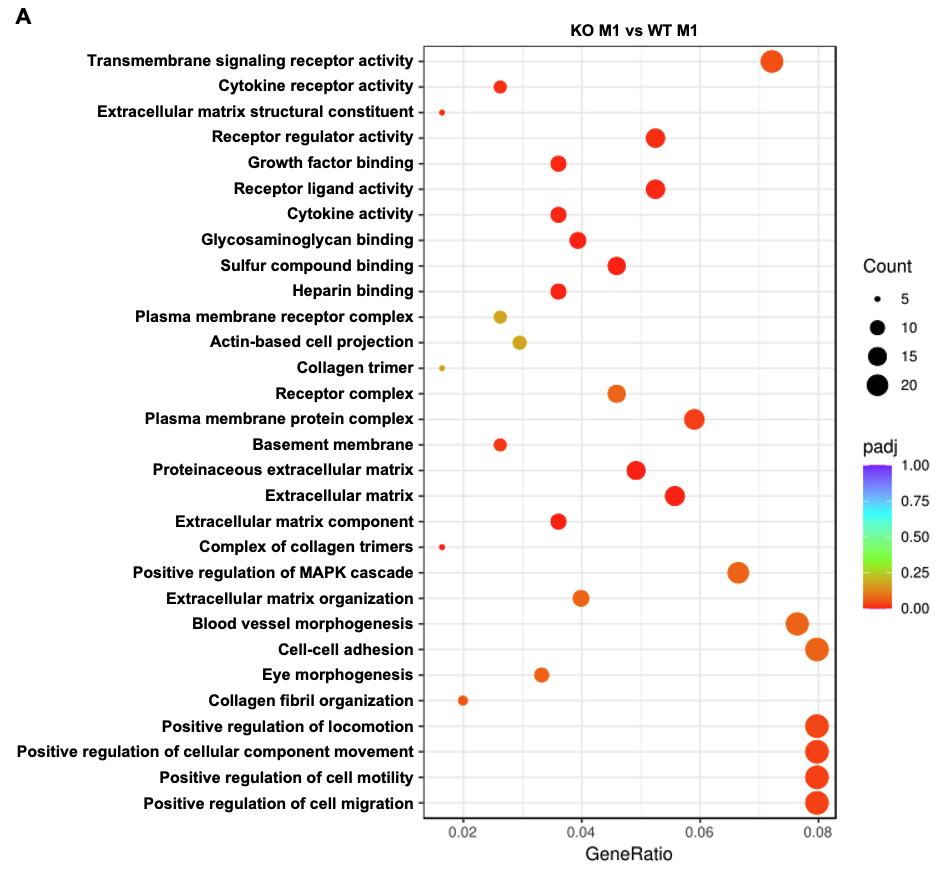


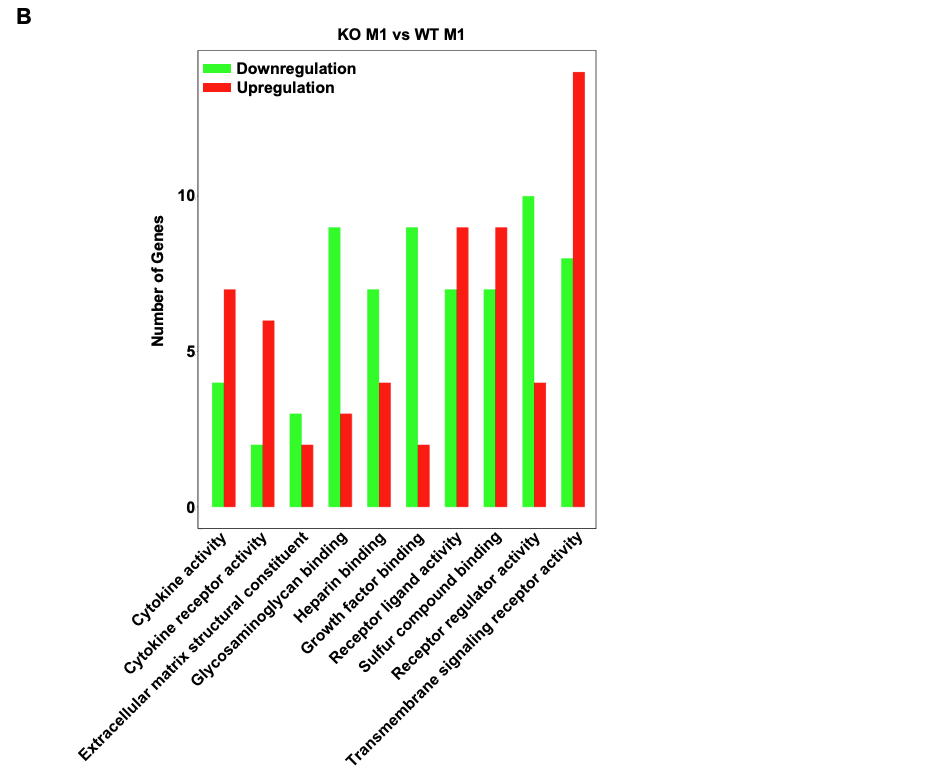


**Fig S5. Gene ontology (GO) enrichment analysis of the differentially expressed genes upon knockout of FRβ.** Bone marrow cells from WT or FRβ KO C57BL/6 mice were differentiated into M0 macrophages and then polarized to M1-like macrophages, as described in Methods. The cells were then used for RNA-sequencing. (**A**) Dot plot showing the top 30 GO enriched functional groups. (**B**) Bar graph showing the changes in the top 10 molecular functional groups (MF) with the highest number of genes upregulated or downregulated in the FRβ KO compared to WT macrophages.


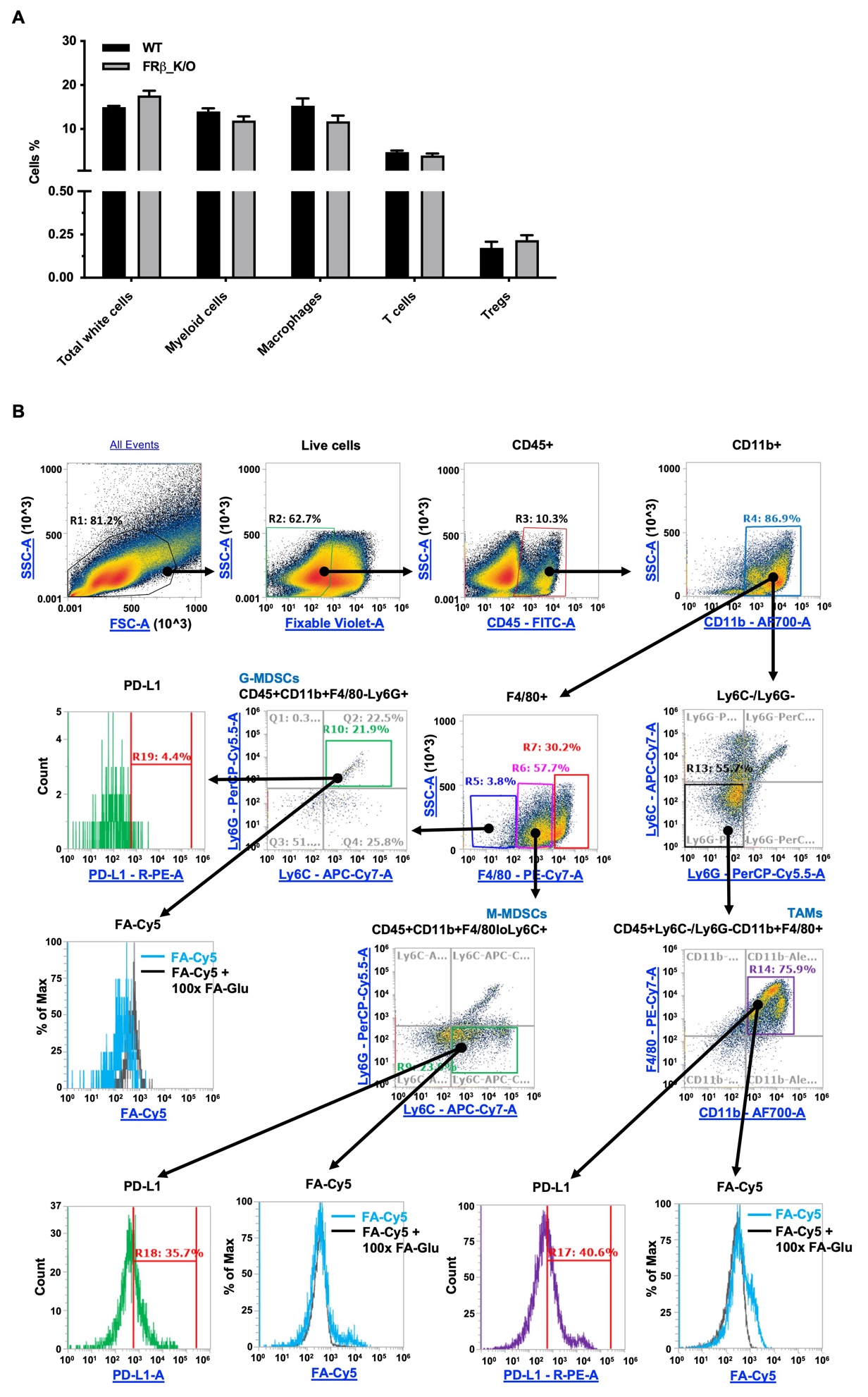


**Fig S6. Immune cells in TRAMP C2 tumors from FRβ KO and WT mice.** TRAMP C2 tumors were grown and harvested from WT or FRβ KO C57BL/6 mice, dissociated in single cells, and stained for flow cytometry analyses as described in Methods. The white cells (CD45+), myeloid cells (CD45+CD11b+), macrophages (CD45+F4/80+), Tregs (CD45+FoxP3CD4+CD25+) and T cells (CD45+CD3+) were observed at similar levels in WT and KO tumors (**A**). Flow cytometry gating strategy for TAMs and MDSCs from the TRAMP C2 tumors (**B**). The CD45+CD11b+F4/80+Ly6C-/Ly6G- cells were considered as TAMs, while CD45+CD11b+Ly6C+ or Ly6G+ cells were considered as MDSCs to quantitate the PD-L1+ TAMs and MDSCs.


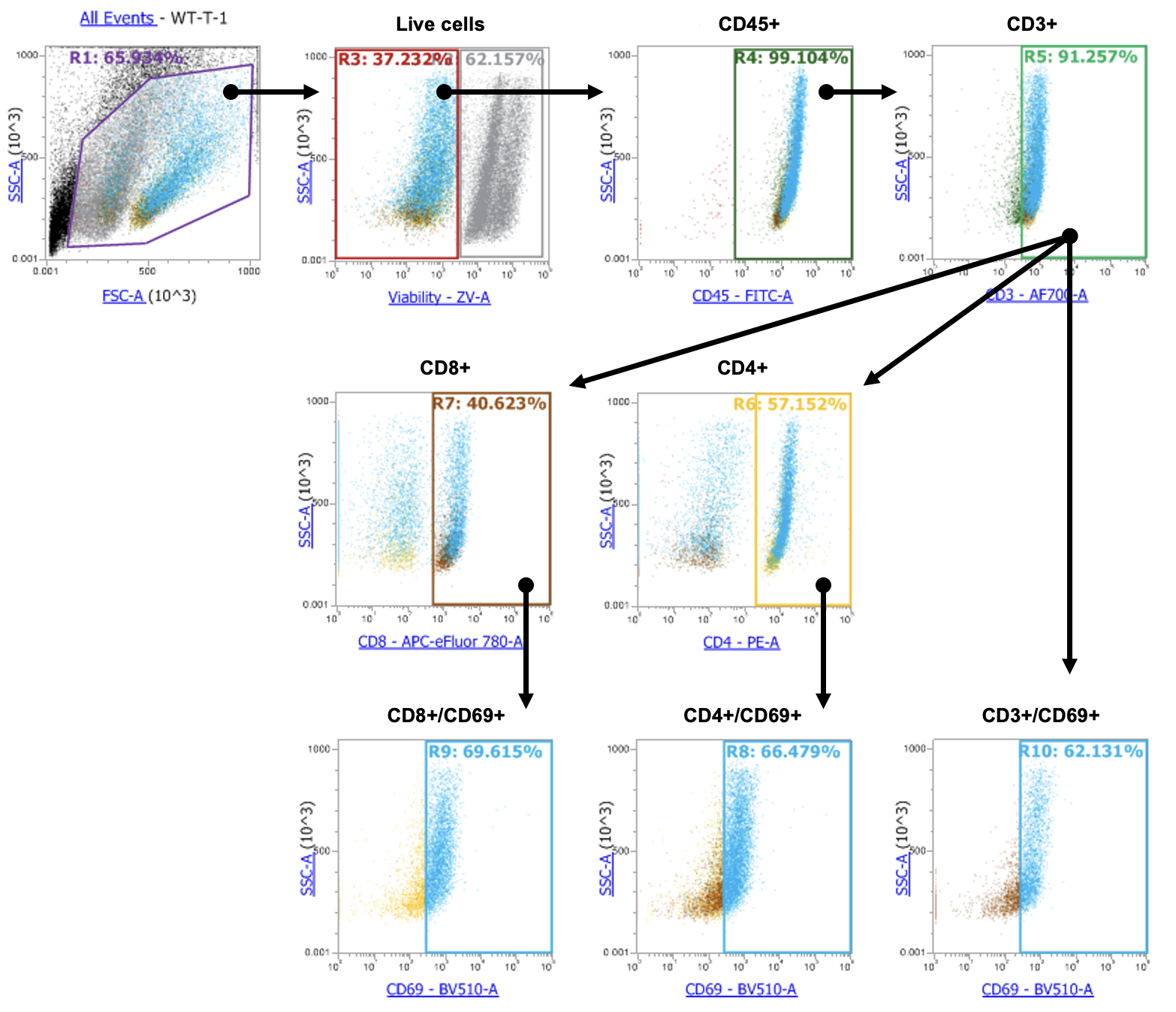


**Fig S7. Flow cytometry gating strategy for the T cell suppression assay.** Healthy M2-macrophages from WT or FRβ KO C57BL/6 mice were co-cultured with activated T cells (harvested from healthy WT C57BL/6 mice and activated as described in Methods) in the presence or absence of mouse anti-FR-β monoclonal antibody (F3 mAb). After co-incubating the cells in the presence of absence of F3 mAb for 18 hours, the CD45+/CD3+/CD69+, CD45+/CD3+/CD4+/CD69+, and CD45+/CD3+/CD8+/CD69+ were considered as CD69+ total T cells, CD69+ CD4+ T cells, and CD69+ CD8+ T cells, respectively for flow cytometry analyses.

**
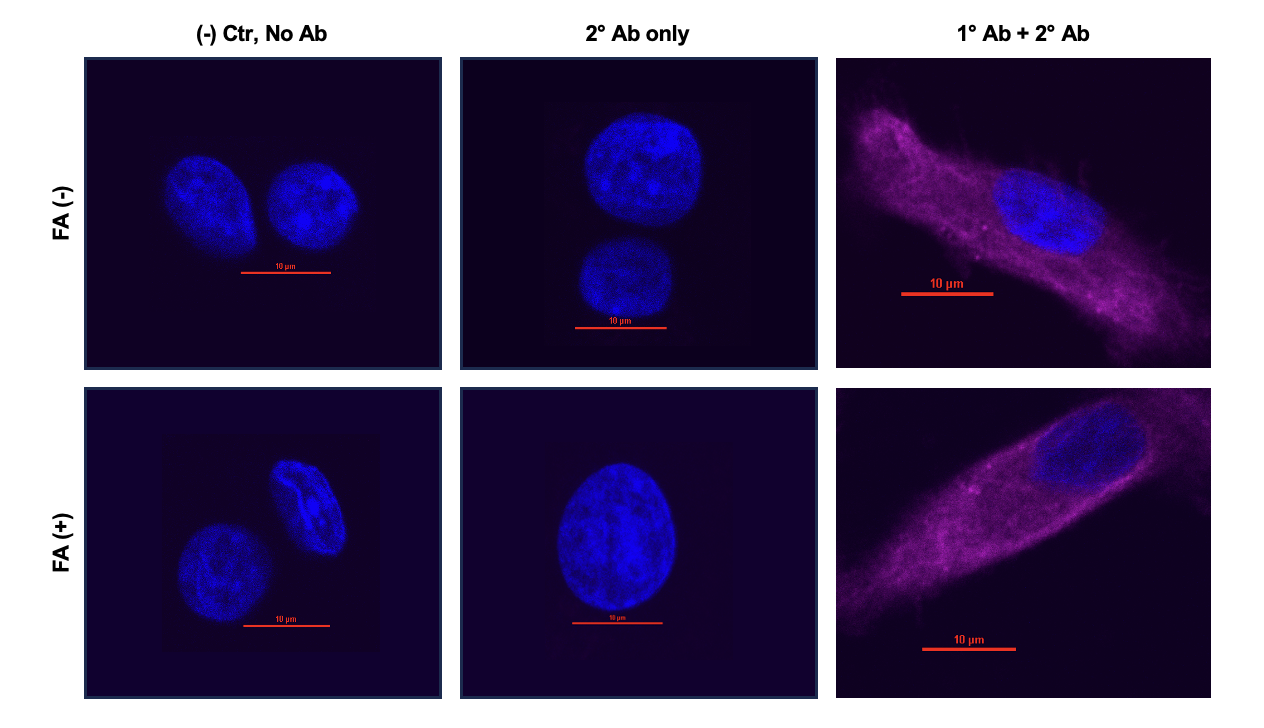
**

**Fig S8. Localization of FRβ in human primary M2-like macrophages in the presence or absence of folic acid.** Human blood derived M2-like macrophages were differentiated, stained, and imaged as described in Methods. Blue, Hoechst 33342 (nuclear stain); Pink, human FRβ monoclonal antibody (m909) with AF488 conjugated secondary antibody. Folate-linked glucosamine (200nM) was used as a control to stimulate internalization of FRβ before antibody staining.


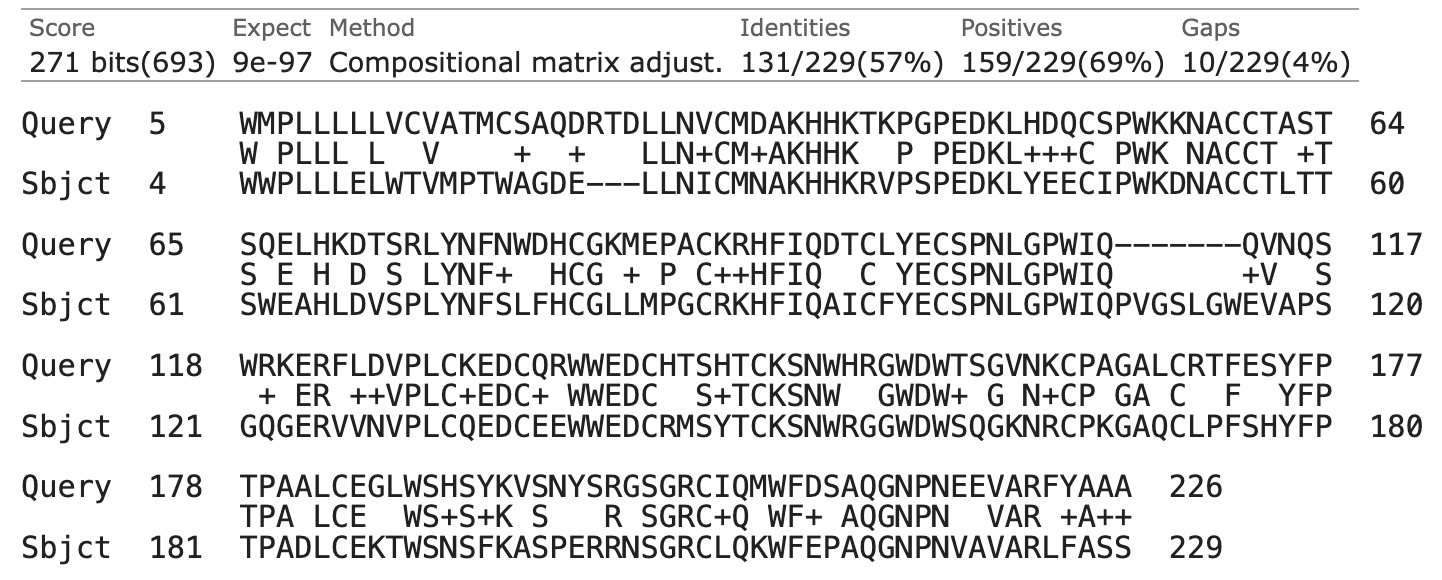


**Fig S9. Sequence alignment between human FRβ and FRδ proteins.** BLAST (blastp) result showed 57.21% Identity between human FRβ and FRδ proteins. Query and Sbjct represents human FRβ and FRδ proteins, respectively.
