# Supplementary material for: Folate receptor β performs an immune checkpoint function in activated macrophages
Source: Front Immunol. 2025 Sep 29;16:1638907. doi: 10.3389/fimmu.2025.1638907 (PMC12515634; doi:10.3389/fimmu.2025.1638907)
Supplement: Supplementary Table 1 — Antibodies and small molecule reagents for flow cytometry. [file Table1.docx]

**Supplementary Tables**

**Table S1: Antibodies and small molecule reagents for flow cytometry**

| Antibody/small molecule reagent | Catalog number | Vendor |
| --- | --- | --- |
| Anti-mouse CD45-FITC | 103107 | Biolegend |
| Anti-mouse CD45-BV510 | 103137 |  |
| Anti-mouse/human CD11b-AF700 | 101222 |  |
| Anti-mouse F4/80-PE/Cy7 | 157307 |  |
| Anti-mouse Ly6C-APC/Cy7 | 128025 |  |
| Anti-mouse Ly6G-PerCP-Cy5.5 | 127615 |  |
| Anti-mouse PD-L1-PE | 155403 |  |
| Anti-mouse CD64-AF647 | 139321 |  |
| Anti-mouse CD3-APC | 100235 |  |
| Anti-mouse CD69-BV510 | 104531 |  |
| Anti-mouse PD1- PE/Cy7 | 109109 |  |
| Anti-mouse CD3-AF700 | 100216 |  |
| Anti-mouse CD4-PE | 100407 |  |
| Anti-mouse CD8-APC-eFluor 780 | 47-0081-82 | Invitrogen |
| FA-Cy5 | NA | Synthesized in-house (Ref: *Front. Immunol*. 15:1354735.) |
| FA-Glucosamine | NA |  |

**Table S2. PCR primer sequences**

| Murine WT FRβ | forward | 5’-TGTGTGAGGCAGGGAAGAAGTG-3’ |
| --- | --- | --- |
|  | reverse | 5’-TGGCTGGTGTTGACTGAGCAA-3’ |
| Murine mutant FRβ | forward | 5’-ATCGCCTTCTATCGCCTTCTTGA-3’ |
|  | reverse | 5’-TGGCTGGTGTTGACTGAGCAA-3’ |
| Murine *Slc19a1* (RFC1) | forward | 5’-GTGACCTTTGTGCTTTTCCG-3’ |
|  | reverse | 5’-TGTGATACAGGTCTTAAGCGC-3’ |
| Murine *Slc46a1* (PCFT) | forward | 5’-TTCCTGTCATTGGTCACGAC-3’ |
|  | reverse | 5’-CGGGTAGATGGAGTTGAAGATG-3’ |
| Murine *Arg1* | forward | 5’-AAGAATGGAAGAGTCAGTGTGG-3’ |
|  | reverse | 5’-GGGAGTGTTGATGTCAGTGTG-3’ |
| Murine *Cd206* | forward | 5’-ATGGATGTTGATGGCTACTGG-3’ |
|  | reverse | 5’-TTCTGACTCTGGACACTTGC-3’ |
| Murine *Mmp9* | forward | 5’-GATCCCCAGAGCGTCATTC-3’ |
|  | reverse | 5’-CCACCTTGTTCACCTCATTTTG-3’ |
| Murine *Nos2* (iNOS) | forward | 5’-GCAAACATCACATTCAGATCCC-3’ |
|  | reverse | 5’-TCAGCCTCATGGTAAACACG-3’ |
| Mouse *Actb* (β-Actin) | forward | 5’-ACCTTCTACAATGAGCTGCG-3’ |
|  | reverse | 5’-CTGGATGGCTACGTACATGG-3’ |
